# SParSE++: improved event-based stochastic parameter search

**DOI:** 10.1186/s12918-016-0367-z

**Published:** 2016-11-25

**Authors:** Min K. Roh, Bernie J. Daigle

**Affiliations:** 1 Applied Mathematics, Institute for Disease Modeling, 3150 139th Ave SE, Bellevue, 98005 WA USA; 2 Departments of Biological Sciences and Computer Science, The University of Memphis, 3744 Walker Avenue, Memphis, 38152 TN USA

**Keywords:** Stochastic simulation, Parameter estimation, Rare event, Optimization, Stochastic event, Stochastic mass action kinetics

## Abstract

**Background:**

Despite the increasing availability of high performance computing capabilities, analysis and characterization of stochastic biochemical systems remain a computational challenge. To address this challenge, the Stochastic Parameter Search for Events (SParSE) was developed to automatically identify reaction rates that yield a probabilistic user-specified event. SParSE consists of three main components: the multi-level cross-entropy method, which identifies biasing parameters to push the system toward the event of interest, the related inverse biasing method, and an optional interpolation of identified parameters. While effective for many examples, SParSE depends on the existence of a sufficient amount of intrinsic stochasticity in the system of interest. In the absence of this stochasticity, SParSE can either converge slowly or not at all.

**Results:**

We have developed SParSE++, a substantially improved algorithm for characterizing target events in terms of system parameters. SParSE++ makes use of a series of novel parameter leaping methods that accelerate the convergence rate to the target event, particularly in low stochasticity cases. In addition, the interpolation stage is modified to compute multiple interpolants and to choose the optimal one in a statistically rigorous manner. We demonstrate the performance of SParSE++ on four example systems: a birth-death process, a reversible isomerization model, SIRS disease dynamics, and a yeast polarization model. In all four cases, SParSE++ shows significantly improved computational efficiency over SParSE, with the largest improvements resulting from analyses with the strictest error tolerances.

**Conclusions:**

As researchers continue to model realistic biochemical systems, the need for efficient methods to characterize target events will grow. The algorithmic advancements provided by SParSE++ fulfill this need, enabling characterization of computationally intensive biochemical events that are currently resistant to analysis.

**Electronic supplementary material:**

The online version of this article (doi:10.1186/s12918-016-0367-z) contains supplementary material, which is available to authorized users.

## Background

The ever-increasing computational capacity of modern computer architectures [1–5] has enabled the simulation of realistic biochemical models with thousands of reactions [6–8]. Despite this capability, computational analysis of stochastic biochemical systems remains a challenge, as techniques like parameter estimation and sensitivity analysis typically require the simulation of multiple ensembles of hundreds to thousands of system trajectories. Most existing parameter estimation algorithms for biochemical systems identify one or more sets of reaction rate parameters giving rise to trajectories that closely mimic observed data [9–15]. In contrast, Stochastic Parameter Search for Events (SParSE) [16] was developed to efficiently sample biochemical reaction rate parameter values that confer a user-specified target event with a given probability and error tolerance. Its conception was inspired by acknowledging the usefulness of such an event-based approach to parameter estimation. As executing SParSE with different initial conditions will identify non-overlapping sets of parameter values that satisfy the target event equally well (the “solution hypersurface”), these results can be used to evaluate various cost functions for scientific and economic purposes. For example, many intervention strategies exist in malaria control: mass drug administration, mass screen and treat, focal mass drug administration, and snowball reactive case detection [17]. Knowing all combinations of system parameters in an epidemiological model that result in eradication of malaria is extremely beneficial for making the most cost-effective policy decisions. Similarly, learning different parameter combinations that result in cell polarization in a mechanistic model of the yeast *Saccharomyces cerevisiae* can aid in our understanding of cell polarization in other organisms as well as contribute new insights to yeast polarization [18]. In general, SParSE outputs from the solution hypersurface may be filtered using user-defined cost functions or constraints to further refine the event characterization.

SParSE is comprised of three main components: the multi-level cross-entropy (CE) method, exponential parameter interpolation, and the inverse biasing method (see below for details) [16]. As introduced in [19], performance of the multi-level CE method depends on the existence of a sufficient amount of intrinsic stochasticity in the system of interest. For systems with low levels of intrinsic stochasticity (even for a subset of reactions), the multi-level CE method can exhibit slow convergence properties, especially when initial parameter values are far from the solution hypersurface. Furthermore, experiments conducted in [16] demonstrated that the accuracy of exponential interpolation significantly decreases when parameter estimates rapidly pass through the solution hypersurface. Taken together, these two limitations greatly hamper our ability to characterize target events in important classes of stochastic systems.

To overcome these limitations, we developed SParSE++, a substantially improved algorithm for characterizing target events in terms of system parameters. SParSE++ makes use of several algorithmic improvements to SParSE that lead to faster and more accurate performance, particularly in the presence of low intrinsic system stochasticity. SParSE++ utilizes a novel method called cross-entropy leaping (CE leaping) that accelerates the convergence rate of the multi-level CE method. Upon detecting slow convergence, CE leaping uses past parameter estimates to intelligently “leap" forward in parameter space rather than continue using the standard CE method. Furthermore, we have defined special leaping cases to improve accuracy in situations when parameter estimates rapidly pass through the solution hypersurface. Finally, SParSE++ features a more robust parameter interpolation method that further accelerates the algorithm convergence rate. To demonstrate superior performance of SParSE++, we apply the method to the three example systems featured in [16]: a birth-death process, a reversible isomerization model, and a system exhibiting SIRS disease dynamics. In addition, we include an eight-reaction model of yeast polarization featured in [13, 20]. In each example, SParSE++ shows substantially improved computational efficiency over SParSE, with the largest efficiency improvements resulting from analysis of events with the strictest error tolerances.

## Methods

The algorithms developed in this work make use of Gillespie’s stochastic simulation algorithm (SSA) [21]—a Monte Carlo simulation method that produces exact trajectories of a well-stirred system obeying the chemical master equation (CME). Such systems can be described in the following manner. Given a biochemical system consisting of *N* molecular species {*S*_1_,⋯,*S*_*N*_} and *M* reaction channels {*R*_1_,⋯,*R*_*M*_}, let *X*_*i*_(*t*) denote the population of *S*_*i*_ at time *t*, **x**(*t*)≡(*X*_1_(*t*),⋯,*X*_*N*_(*t*)) the state vector at time *t*, and **x**_0_≡**x**(*t*_0_) the population at initial time *t*_0_. The time evolution of **x**(*t*) in a fixed volume at constant temperature is governed by sequences of two random variables: *τ*, the time elapsing between the current and next reaction firings, and *j* ^′^, the index of the next reaction firing at time *t*+*τ*. After each selection of *τ* and *j* ^′^, **x**(*t*) advances by $\mathbf {x}(t+\tau) = \mathbf {x}(t) + \boldsymbol {\nu }_{j^{\prime }}$, where ***ν***_*j*_≡[*ν*_1*j*_,⋯,*ν*_*Nj*_], and *j*∈{1,⋯,*M*} is the state change (stoichiometry) vector. Each component in the state change vector, *ν*_*ij*_, denotes the change in population *X*_*i*_ induced by single firing of reaction *R*_*j*_.

Sampling *τ* and *j* ^′^ requires computation of reaction propensity functions, *a*_*j*_(**x**,**k**), where **k** represents the system reaction rates (*k*_1_,⋯,*k*_*M*_), defined such that *a*_*j*_(**x**,**k**)d*t* is the probability that one *R*_*j*_ reaction occurs in the next infinitesimal time interval [*t, t*+d*t*). Denoting the propensity sum as $a_{0}(\mathbf {x}, \mathbf {k}) \equiv \sum _{j=1}^{M} a_{j}(\mathbf {x}, \mathbf {k})$, each time to the next reaction *τ* is exponentially distributed with mean 1/*a*_0_(**x**,**k**), and each index of the next reaction *j* ^′^ is categorically distributed with probability *a*_*j*_(**x**,**k**)/*a*_0_(**x**,**k**),*j*∈{1,⋯,*M*}.

Our goal in simulating system trajectories is to characterize the probability of reaching a target event $\mathcal {E}$ before some final time *t*_*f*_. Thus, during simulation we update each trajectory’s state until either *t*_*f*_ is reached or the target event $\mathcal {E}$ occurs (denoted by stopping time $\mathcal {T}$). After simulating *N*_*S*_ trajectories, the Monte Carlo estimate for $\mathcal {E}$ can be expressed as

1 $$ \hat{p}(\mathbf{x}_{0}, \boldsymbol{k}, \mathcal{E}; t_{f}) = \frac{1}{N_{S}} \sum_{i=1}^{N_{S}} \left[ I_{\{f(\mathbf{x}_{i}(\boldsymbol T_{i} | \boldsymbol{k})) \cap \mathcal{E}\}} \right],  $$

where *f*(**x**_*i*_(***T***_*i*_|***k***)) is the value of the event function *f*(·) evaluated on the *i* ^th^ trajectory at times $\boldsymbol T_{i} \equiv \{t_{0}, t_{i1}, \cdots, t_{iN_{\mathcal {T}_{i}-1}}, \mathcal {T}_{i}\}$ (*t*_*ik*_ is the firing time of the *k* ^th^ reaction and $N_{\mathcal {T}_{i}}$ is the number of reaction firings occurring in the *i* ^th^ trajectory) simulated with reaction rates ***k***. Two requirements for *f*(·) are that it takes the system state as an input and can be used to evaluate the distance between this state and $\mathcal {E}$. The indicator function $I_{\{f(\mathbf {x}_{i}(\boldsymbol {T}_{i} | \boldsymbol {k})) \cap \mathcal {E}\}}$ thus returns a value of 1 if the distance between *f*(**x**_*i*_(***T***_*i*_|***k***)) and $\mathcal {E}$ is zero and 0 otherwise.

### SParSE—stochastic parameter search for events

In this section, we briefly describe the original SParSE algorithm. We refer the reader to [16] for details concerning the algorithm. The objective of SParSE is to find reaction rates ***k*** ^∗^ that satisfy

2 $$ \left|\mathcal{P}_{\mathcal{E}} - \hat{p}(\mathbf{x}_{0}, \boldsymbol{k}^{*}, \mathcal{E}; t_{f})\right| \leq \epsilon_{\mathcal{P}_{\mathcal{E}}},  $$

where $\mathcal {P}_{\mathcal {E}}$ and $\epsilon _{\mathcal {P}_{\mathcal {E}}}$ are the user-defined target probability of observing event $\mathcal {E}$ by time *t*_*f*_ and user-defined absolute error tolerance, respectively. Starting with ***γ*** ^0^≡***1*** and ***k*** ^0^, SParSE advances the system toward $\mathcal {E}$ by iteratively updating ***k*** ^(*l*)^ by

3 $$ k^{(l)}_{j} = k^{(l-1)}_{j} \times \gamma^{(l)}_{j}, \ j \in [1,\cdots,M].  $$

The multi-level cross-entropy (CE) biasing parameters ***γ*** ^(*l*)^ are computed by

4 $$ \gamma_{j}^{(l)} = \frac{{\sum}_{i}' n_{ij}}{{\sum}_{i}' {\sum}_{k=1}^{N_{{\mathcal{T}}_{i}}} \left[ a_{j}(\mathbf{x}_{i}(t_{ik}), \boldsymbol{k}^{(l-1)}) \tau_{ik} \right]} \;,  $$

where *n*_*ij*_ is the total number of times reaction *j* fires in the *i* ^th^ trajectory, $\sum _{i}'$ iterates only over the subset of *N*_*S*_ trajectories that return 1 for $I_{\{f(\mathbf {x}_{i}(\boldsymbol {T}_{i} | \boldsymbol {k})) \cap \mathcal {E}\}}$, *k* indexes the $N_{\mathcal {T}_{i}}$ reaction firings occurring in the *i* ^th^ trajectory, and *t*_*ik*_ and *τ*_*ik*_ represent the absolute time and time elapsed since the last firing for the *k* ^th^ reaction firing in the *i* ^th^ trajectory. Computation for ***γ*** ^(*l*)^ and ***k*** ^(*l*)^ terminates when Eq. (2) is satisfied, or when $l \geq \mathcal {L}$ for some $\mathcal {L} \in \mathbb {N}$ (10 by default).

Besides the multi-level CE method, SParSE is comprised of two other components: exponential parameter interpolation and the inverse biasing method. Both the multi-level CE and the inverse biasing methods proceed by picking a set of intermediate events ***ξ*** that are close to $\mathcal {E}$ and reachable with the current reaction rates. SParSE chooses ***ξ*** at each iteration by selecting the top ⌈***ρ*** *N*_*S*_⌉ simulated trajectories that evolve farthest in the direction of $\mathcal {E}$. The values of ***ρ*** are chosen based on the distance between the current estimate and $\mathcal {P}_{\mathcal {E}}$. Denoting the distance as $\delta (\boldsymbol {k}) \equiv \mathcal {P}_{{E}} - \hat {p}(\mathbf {x}_{0}, \boldsymbol {k}^{(l-1)}, \mathcal {E}; t_{f})$, ***ρ*** is chosen by

For sgn(*δ*(***k***))==*ϕ*_type_

5 $$ \boldsymbol{\rho}(\delta) = \left\{ \begin{array}{ll} \left[0.005, 0.01\right] & \text{if}~0.4 < |\delta| \\ \left[0.01, 0.05, 0.1\right] & \text{if}~0.2 <|\delta| \leq 0.4\\ \left[0.05, 0.1, 0.2\right] & \text{otherwise} \end{array}\right.  $$

For sgn(*δ*(***k***))≠*ϕ*_type_ (i.e., over-perturbation)

6 $$ \boldsymbol{\rho}(\delta) = \left\{\begin{array}{ll} [0.01, \ 0.015] & \text{if}~ 0.4~< |\delta| \\ \left[0.05, \ 0.1, \ 0.15\right] & \text{if}~0.2 < |\delta| \leq 0.4\\ \left[0.1, \ 0.15, \ 0.2\right] & \text{otherwise}, \end{array}\right.  $$

where *ϕ*_type_ is 1 if $f(\mathbf {x}_{0}) \leq \mathcal {E}$ and -1 otherwise. In the above two cases, sgn(*δ*(***k***))≠*ϕ*_type_ corresponds to the case where the current reaction rates over-perturb the system with respect to $\mathcal {P}_{\mathcal {E}}$. The conventional multi-level CE method cannot be used here, as the top trajectories evolving in the direction of $\mathcal {E}$ surpass the target event more than the desired amount. These trajectories, however, can be used to reverse the direction of bias (thus the term inverse biasing). Instead of terminating a simulated trajectory when $\mathcal {E}$ is reached (as is done in the multi-level CE method), we run all *N*_*S*_ trajectories until *t*_*f*_ and record the maximum values reached in the direction of the target event. These values are then used in Eq. (6) to determine intermediate target events. As with Eq. (5), the smaller the distance, the less extreme intermediate events are chosen to avoid excessive biasing. The inverse of the biasing parameters corresponding to these events are then multiplied by the current reaction rates to compute the next estimates $\hat {p}$. While inverse biasing effectively reverses the direction of bias in the case of over-perturbation, estimates computed in this way may not be accurately characterized by exponential interpolation. Thus, we choose more conservative ***ρ*** values for inverse biasing than for the multi-level CE method.

For target events that require high accuracy (i.e., small user-defined error tolerance), the multi-level CE method may ‘step over’ ***k*** ^∗^, resulting in both under- and over-perturbing ***γ*** values. In this case, SParSE performs fine-tuning by exponential interpolation, which computes parameters ***q*** and ***r*** that satisfy

7 $${} \min_{q_{j},r_{j}} \left(\!(\boldsymbol{\hat{p}}^{(\cdot)} \times N_{S}) - q_{j} \times \text{exp}\left\{r_{j} \times \gamma_{j}^{(0,\cdot)} \!\right\} \right), \ j=\{1,\cdots,M\},  $$

where ***γ*** ^(0,·)^ are the values of the past multi-level CE method biasing parameters normalized with respect to ***γ*** ^0^. Denoting ***k*** ^(*l*)^ as the reaction rates for the current iteration of SParSE, each $ \gamma ^{(0, l)}_{j}$ can be expressed as $k^{(l)}_{j} / {k^{0}_{j}}$. The resulting estimate from employing ***γ*** ^(0,*l*)^ is denoted $\hat {p}^{(l)}$. Since interpolation is initiated only after both under- and over-perturbing estimates are obtained, ***γ*** ^(0,·)^ is guaranteed to have at least two entries. When there are more than five entries, SParSE picks the five estimates closest to $\mathcal {P}_{\mathcal {E}}$ while requiring that both under- and over-perturbed values are present. Once the optimal exponential curve in Eq. (7) is found, SParSE returns up to seven sets of candidate biasing parameters. Three of the seven correspond to estimates that are slightly less than $\mathcal {P}_{\mathcal {E}} \times N_{S}$, one corresponds to the exact value, and the rest to estimates slightly greater than $\mathcal {P}_{\mathcal {E}} \times N_{S}$. Interpolation starts with the candidate set from the exact target value, and it shifts to over- or under-perturbing parameters depending on the resulting estimate. For example, if the resulting estimate is greater than $\mathcal {P}_{\mathcal {E}} \times N_{S} + \epsilon _{\mathcal {P}_{\mathcal {E}}}$, SParSE chooses the next under-perturbing biasing parameters to compute the next estimate. SParSE assumes failure in interpolation and exits if it is unable to find ***k*** ^∗^ in a user-defined number of iterations, $\mathcal {I}$, which is set to 3 by default.

### Accelerating convergence with cross-entropy leaping

We now describe the enhancements made to create SParSE++, a substantially improved algorithm for identifying ***k*** ^***∗***^ for a given event and target probability. The first enhancement introduces cross-entropy (CE) leaping, an algorithmic technique enabling accelerated convergence.

Given a set of initial reaction rates, the SParSE convergence rate depends on the intrinsic stochasticity of the system with respect to the target event, which we simply refer to as ‘stochasticity’ in the remainder of this section. Denoting $\xi _{i}^{\text {max}}$ as the value of *f*(**x**_*i*_(*t*|***k*** ^(*l*)^)) closest to $\mathcal {E}$ reached by the *i* ^th^ trajectory, the next intermediate events ***ξ*** are chosen as the closest ⌈***ρ*** *N*_*S*_⌉ values of $\xi _{i}^{\text {max}}$ to $\mathcal {E}$, where ***ρ*** is chosen by (5) or (6) and *i*∈{1,⋯,*N*_*S*_}. For systems and target events exhibiting low stochasticity, the variance among the $\xi _{i}^{\text {max}}$ values is small. As a result, even small values of ***ρ*** will generate subsequent intermediate events that are very close to current ones, causing SParSE to converge slowly, if at all.

To accelerate convergence for low stochasticity cases, we developed a method called cross-entropy (CE) leaping that computes ***γ*** using exponential extrapolation from past biasing parameters and probability estimates. Starting with *l*=1, SParSE++ records $\hat {p}(\mathbf {x}_{0}, \boldsymbol {k}^{(l)}, \mathcal {E}; t)$ and initiates CE leaping if neither of the following two conditions are true:

1. Inequality (2) is satisfied. In this case the objective of SParSE++ is met, and the algorithm exits,
2. $ \left \{\begin {array}{lr} \hat {p}\cdot N_{S} \leq \mathcal {P}_{\mathcal {E}}\cdot N_{S} \cdot 0.01, & \text {for} \mathcal {P}_{\mathcal {E}} \leq 0.5\\ \hat {p}\cdot N_{S} \leq (1 - \mathcal {P}_{\mathcal {E}})\cdot N_{S} \cdot 0.01, & \text {for} 0.5 < \mathcal {P}_{\mathcal {E}} \end {array}\right \} $

Condition 2 is enforced to ensure the signal from ***k*** ^(*l*)^ is reliable; at least 1*%* of the fraction of trajectories equal to $\mathcal {P}_{\mathcal {E}}$ (or $1-\mathcal {P}_{\mathcal {E}}$) are required in order to qualify for leaping. SParSE++ repeatedly clears the memory of past estimates until two qualifying probability estimates are observed consecutively, at which point Algorithm 1 is executed to determine leaping eligibility and magnitude.



Here, input variables $\hat {\boldsymbol p}$ and ***γ*** denote consecutive probability estimates and their corresponding biasing parameters, respectively. CE leaping utilizes the mean rate of convergence calculated from $\hat {\boldsymbol p}$ to determine how far in biasing parameter space to leap forward (Line 13). To compute the convergence rate of the first probability estimate, we include the estimate computed immediately before the first eligible $\hat {p}$ when possible. The only instance when this cannot be done is when two eligible values from $\hat {\boldsymbol p}$ are the very first two estimates computed for a given ***k*** ^0^.

The states from which CE leaping are triggered can vary greatly. For example, the distance to the target probability $\mathcal {P}_{\mathcal {E}}$, rate of change in $\hat {\boldsymbol p}$, and the magnitude of $\mathcal {P}_{\mathcal {E}}$ can all differ substantially, even for the same systems using different values of ***k*** ^0^. The amount of leaping used should thus depend on all of these factors, in order to avoid grossly over-perturbing the system. To handle different rates of convergence, two pre-defined variables leap_projs_ and leap_factors_ are used to adjust the amount of leaping based on the average change in $\hat {\boldsymbol p}\!\cdot \!N_{S}$ (mean_step_) and the minimum distance to the target event probability (dist_min_). The largest leaping multiplier (7 from Line 4) is chosen when the estimated number of steps to reach $\mathcal {P}_{\mathcal {E}}$ (step_proj_; Line 13), is greater than 50 (Line 3). In contrast, when step_proj_ is less than 5 the standard multi-level CE method is used instead of CE leaping. The value for step_proj_ is computed assuming linear convergence with rate mean_step_. As this assumption may not be valid for certain systems and target event functions *f*(**x**), leaping multipliers (leap_factors_ in Line 4) are chosen conservatively to prevent over-perturbation.

When CE leaping is triggered, SParSE++ skips the computation of intermediate target events and their associated biasing parameters, as ***γ*** ^(*l*)^ is set to ***γ*** ^CE^.

### Special leaping cases

The CE leaping algorithm is designed to accelerate the rate of convergence of the multi-level CE method when it is stuck on a “plateau” in parameter space. The opposite scenario can also pose a problem to SParSE—in a “steep” region of parameter space, the multi-level CE method may pass through the solution hypersurface too quickly. This can lead to erroneous interpolants or even solution divergence when computed estimates do not meet the thresholds required for interpolation and leaping. Three cases of this scenario are identified and handled in SParSE++. Starting from the least severe instance and moving to the most severe, we explain each case in detail. As with CE leaping, new biasing parameters are computed from past estimates; thus, description of the second stage of the multi-level CE method (i.e., ***γ*** computation based on intermediate target events) is omitted below.

#### Last leaping prior to interpolation

In a quickly-changing parameter region, SParSE may enter the interpolation stage with as few as two estimates, one on either side of $\mathcal {P}_{\mathcal {E}}$. Even when more than two estimates exist, their values may be far from $\mathcal {P}_{\mathcal {E}}$ if only two iterations of the multi-level CE method are run prior to interpolation. Lack of $\hat {p}$ values near $\mathcal {P}_{\mathcal {E}}$ can significantly degrade interpolation quality. This case can be avoided by first obtaining another estimate near $\mathcal {P}_{\mathcal {E}}$. To do this, SParSE++ computes an additional estimate prior to entering the interpolation stage using ***γ*** ^WA^, the values of which are generated using Algorithm 2.



Here *N*_*S*_ and $\mathcal {P}_{\mathcal {E}}$ denote the total number of simulated trajectories and the user-defined target probability, respectively. The least under- and over-perturbing biasing parameters (***γ*** ^*u*^ and ***γ*** ^*o*^) are guaranteed to exist, since Algorithm 2 is run immediately prior to the interpolation stage, which is only triggered when both under- and over-perturbing estimates have been computed. The weights *w* ^*u*^ and *w* ^*o*^ for ***γ*** ^*u*^ and ***γ*** ^*o*^ reflect how close $\hat {p}^{u}$ and $\hat {p}^{o}$ are to $\mathcal {P}_{\mathcal {E}}$. Supposing $\hat {p}^{u}$ is closer to $\mathcal {P}_{\mathcal {E}}$ than $\hat {p}^{o}$, then *δ* ^*u*^ (the distance between $\mathcal {P}_{\mathcal {E}}$ and $\hat {p}^{u}$) will be smaller than *δ* ^*o*^. Since *w* ^*u*^ is defined as *δ* ^*o*^ normalized by the total distance, *w* ^*u*^ will be greater than *w* ^*o*^. Thus, the weighted averaging method gives more weight to biasing parameters from the better estimate. When characterizing target events with a larger error tolerance, the final leaping performed with ***γ*** ^WA^ may satisfy Eq. (2) and thus eliminate the need to run interpolation.

#### Leaping on low-signal region

Near a quickly-changing region of parameter space, one iteration of SParSE++ can alter biasing parameters so much that the next estimate does not qualify for interpolation or CE leaping. In this case, the multi-level CE method may take many iterations to escape this “low-signal” region. Algorithm 2 can also be used to improve performance in this case, provided that all three of the following conditions are met:

1. At least three previous estimates exist
2. At least one estimate is located on either end of $\mathcal {P}_{\mathcal {E}}$
3. At least one estimate on one side of $\mathcal {P}_{\mathcal {E}}$ is eligible for interpolation, and every estimate on the other side does not qualify

The last condition corresponds to the case where estimates on one side of $\mathcal {P}_{\mathcal {E}}$ contain a sufficient signal for interpolation, while estimates on the other side do not. We note that although Algorithm 2 is executed both here and in the previous special case, the conditions that trigger the algorithm as well as its purpose are very different. In the previous section, Algorithm 2 is used to compute an estimate close to the target probability before beginning the interpolation stage. Here, the same method is used to escape a low-signal region in an efficient manner.

#### Bisection to obtain sufficient signal

The final case occurs when all past estimates exhibit insufficient signal for interpolation. Although expected to occur rarely, the multi-level CE method can produce estimates that either reach the target event too few or too many times. If the corresponding under- and over-perturbation are severe, these estimates will not be considered for CE leaping or interpolation. When all recorded estimates do not meet the interpolation threshold yet exist on both sides of the target event probability $\mathcal {P}_{\mathcal {E}}$, SParSE++ executes bisection on previous biasing parameters in an attempt to move the system closer to $\mathcal {P}_{\mathcal {E}}$. Using Algorithm 3, we compute the biasing parameters with bisection, ***γ*** ^BS^.



Unlike Algorithm 2, we do not measure distances of $\hat {p}^{u}$ and $\hat {p}^{o}$ with respect to $\mathcal {P}_{\mathcal {E}}$. Since these estimates exhibit insufficient signal, their distances to the target event probability do not contain useful information for computing weights of ***γ*** ^*u*^ and ***γ*** ^*o*^.

We note that this final case was not observed in any of the examples evaluated in the next section. However, the event functions used with the four example systems are simply the states of a single species. For more complicated event functions, we expect this case to occur more frequently, and Algorithm 3 will thus reduce the incidence of solution divergence.

### Improved interpolation

Exponential interpolation in SParSE plays an integral role when the multi-level CE method alone is unable to deliver the target event probability with acceptable precision. Once both under- and over-perturbed estimates are observed that do not satisfy the desired probability range ($\mathcal {P}_{\mathcal {E}} \pm \epsilon _{\mathcal {P}_{\mathcal {E}}}$), SParSE employs exponential interpolation on previous estimates to compute candidate biasing parameters. Acknowledging that the computed interpolant may still not produce an estimate within the required accuracy, SParSE returns up to seven sets of biasing parameters that correspond to slightly perturbed target event probabilities. Using these candidate parameter sets, SParSE computes the first new estimate using the biasing parameters corresponding to the exact target event probability $\mathcal {P}_{\mathcal {E}}$. If the estimate is too high ($\mathcal {P}_{\mathcal {E}} + \epsilon _{\mathcal {P}_{\mathcal {E}}} < \hat {p}$), SParSE picks a set of candidate biasing parameters corresponding to the least under-perturbing probability to compute the next estimate. If the estimate is too low ($\hat {p} < \mathcal {P}_{\mathcal {E}} - \epsilon _{\mathcal {P}_{\mathcal {E}}}$), SParSE picks the least over-perturbing probability instead. This process continues until ***k*** ^∗^ is found or no more candidate biasing parameters remain, whichever occurs first. The default limit on the number of interpolation rounds is set to 3 (i.e., up to 21 candidate biasing parameter sets are computed). If the algorithm does not find ***γ*** ^∗^ at the end of the third round of interpolation, SParSE assumes failure to converge and exits.

The motivation behind working with multiple candidate biasing parameter sets in the SParSE interpolation stage is that the candidate set corresponding to $\mathcal {P}_{\mathcal {E}}$ may not produce a sufficiently accurate estimate, whereas a set corresponding to interpolant values near $\mathcal {P}_{\mathcal {E}}$ might. Thus, SParSE has as many as six alternate biasing parameter sets to be chosen should the first set fail to satisfy Eq. (2). We note that this approach is only helpful if ***γ*** ^∗^ falls within the range of candidate biasing parameter values. In the worst case when this is not true, SParSE must run four additional SSA ensembles to produce an estimate before computing a new interpolant.

SParSE++ greatly improves the efficiency in this worst case scenario by modifying the process of exponential interpolation. First, it computes up to three different exponential interpolants: one as in SParSE, one without the farthest under-perturbing ***γ*** (when more than two under-perturbed estimates exist), and one without the farthest over-perturbing ***γ*** (when more than two over-perturbed estimates exist). For each reaction, SParSE++ then chooses the interpolant with the highest *R* ^2^ statistic. *R* ^2^ statistics are commonly used to assess goodness of fit of statistical models to observed data [22]. For our purposes, the *R* ^2^ value, which is between 0 and 1, indicates the fraction of the total variance of output ($\hat {p}$) that is explained by variation in input (***γ***). Computing the three interpolants and their corresponding *R* ^2^ statistics incurs a negligible computational cost, as no additional SSA simulations are required. Pseudocode for computing the next reaction rates using SParSE++ exponential interpolation is listed in Algorithm 4.

Unlike in SParSE, the chosen interpolant in SParSE++ returns only a single set of biasing parameters corresponding to the exact value of $\mathcal {P}_{\mathcal {E}}$, and new interpolants are only computed if the corresponding estimate does not satisfy Eq. (2). The computational cost of repeatedly generating new interpolants and *R* ^2^ scores in each stage is trivial compared to the cost of simulating a single SSA trajectory for most systems. If the projected biasing parameters $\bar {\boldsymbol {\gamma }}$ from Algorithm 4 are out of range for any reaction *R*_*j*_, a weighted average is used to replace $\bar {\gamma }_{j}$, where the weights are normalized distances between $\mathcal {P}_{\mathcal {E}}$ and estimates corresponding to the least under- and over-perturbing biasing parameter sets for *R*_*j*_.



## Results and discussion

In this section we compare the performance of SParSE++ to that of SParSE using the same three models—a birth-death process, a reversible isomerization model, and a susceptible-infectious-recovered-susceptible (SIRS) disease transmission system—described in [16], as well as an additional eight-reaction system modeling yeast polarization [20]. For the first three models, all possible combinations of $\mathcal {P}_{\mathcal {E}} \in \{0.40, 0.60, 0.80\}$ and $\epsilon _{\mathcal {P}_{\mathcal {E}}} \in \{0.01, 0.05, 0.10\}$ are analyzed by simulating ensembles of *N*_*S*_=5×10^4^ trajectories. For the birth-death process, we also simulated an ensemble of *N*_*S*_=2×10^5^ for $\mathcal {P}_{\mathcal {E}} = 0.010$ and $\epsilon _{\mathcal {P}_{\mathcal {E}}} = 0.001$ in order to illustrate the robustness of SParSE++ on a low probability target event. Similarly, we explored the high probability target event $\mathcal {P}_{\mathcal {E}} = 0.95$ and $\epsilon _{\mathcal {P}_{\mathcal {E}}} = 0.005$ with the reversible isomerization model using ensemble size *N*_*S*_=10^5^. Lastly, the yeast polarization model is studied with $\mathcal {P}_{\mathcal {E}} = 0.60$, $\epsilon _{\mathcal {P}_{\mathcal {E}}} = 0.01$, and *N*_*S*_=5×10^4^.

In order to minimize output differences resulting from stochasticity (as opposed to methodological differences), we used the same random number generator seeds and initial reaction rates for SParSE and SParSE++. For fairness of comparison, we treat each of the following computations as a single iteration: estimation using the multi-level CE method, computing biasing parameters for intermediate events, estimation using interpolation, and estimation using any type of leaping (CE, weighted average, and bisection). Although the exact costs of these computations differ depending on the values of reaction rates and the type of simulations (e.g., computation of intermediate events without over-perturbation is often cheaper than with over-perturbation), the complexity in terms of the number of trajectories simulated is the same, i.e., $\mathcal {O}(N_{S})$. Overall, this procedure ensures that the net computational gain or loss in terms of the total number of trajectories generated is properly quantified. Using this measure we define $\text {gain}_{i} (\%):= \frac {\text {No.\ Iterations SParSE}-\text {No.\ Iterations SParSE++}}{\text {No.\ Iterations SParSE}} \times 100$ to assess the performance of SParSE++ compared to SParSE for a specific combination of $\mathcal {P}_{\mathcal {E}}$ and $\epsilon _{\mathcal {P}_{\mathcal {E}}}$ values. The numerator is the difference between the number of iterations employed by the two methods, while the denominator is the total number of iterations SParSE required. This fraction is multiplied by 100 to create a percentage. Similarly, we define $\text {gain}_{t} (\%) := \frac {\text {SparSE runtime}-\text {SparSE++ runtime}}{\text {SparSE runtime}} \times 100$ for comparison of absolute runtime (in seconds). Both variables gain_*i*_ and gain_*t*_ measure the relative computational ‘gain’ from using SParSE++ over SParSE. All simulations were run on Intel^®;^ Xeon^®;^ CPU E5-2620 v2 at 2.10 GHz workstation with 16 GB RAM, 64-bit Windows 10 Enterprise OS, using Matlab and its Parallel Computing Toolbox™.

Lastly, we note that SParSE++ achieved 100% success on all examples tested and therefore omit explicitly listing the success rate in any of the tables. For examples where SParSE observed failure [16], we examine the role of new features in SParSE++ that enabled the algorithm to successfully converge to the solution hypersurface.

### Birth-death process

**Fig. 1:**
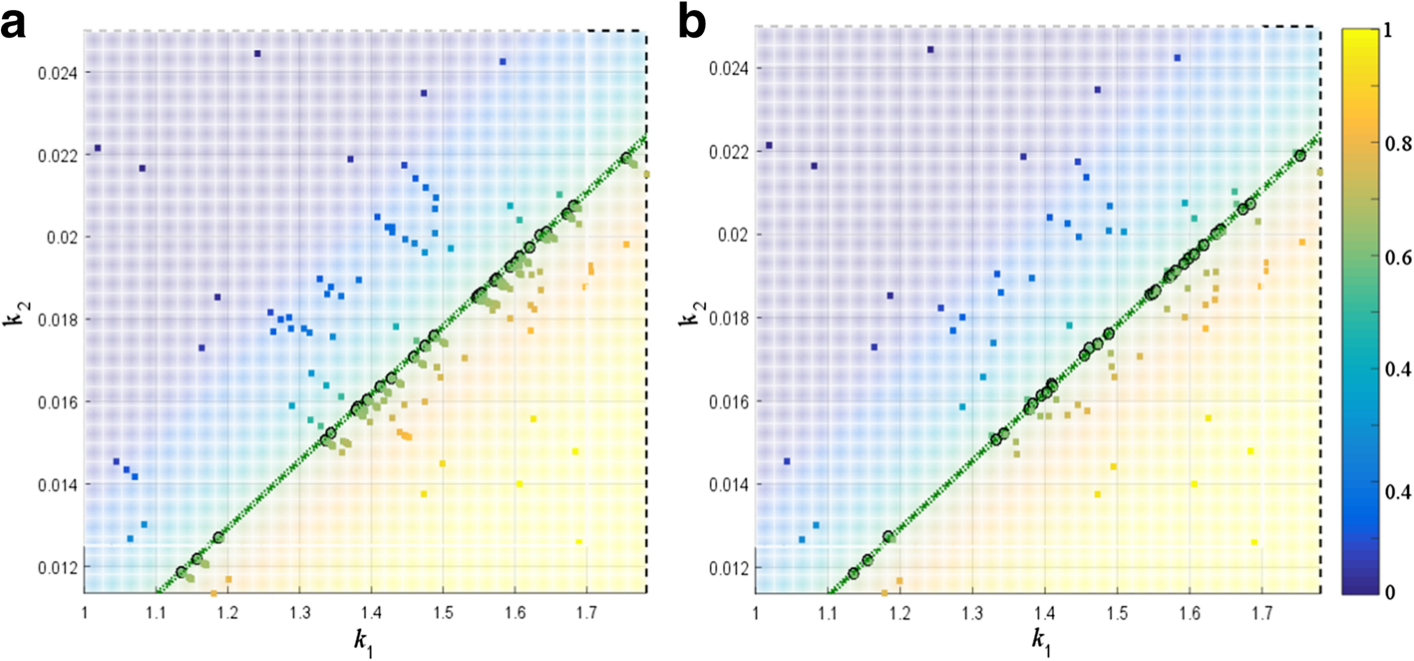
Ensemble result comparison between SParSE **a** and SParSE++ **b** performance for birth-death process with $\mathcal {P}_{\mathcal {E}} = 0.60$ and $\epsilon _{\mathcal {P}_{\mathcal {E}}} = 0.01$. For given thirty initial reaction rates, SParSE required 166 estimates while SParSE++ required 122

Our first example is the birth-death process, which is defined as follows:

$$\begin{array}{*{20}l} \emptyset & \stackrel{k_{1}}{\rightarrow} S,& 1.0 \leq & k_{1} \leq 1.7 \\ S & \stackrel{k_{2}}{\rightarrow} \emptyset, & 0.0125 \leq & k_{2} \leq 0.025,\\ \end{array} $$

with **x**_0_=[40] and $\mathcal {E}$ the population of *S* reaching 80 before *t*_*f*_=100. Table 1 summarizes the results for the 10 test cases. We note that SParSE++ attained 100% convergence as well as significant computational gains for problems that required high accuracy. Figure 1 illustrates ensemble results from running SParSE (a) and SParSE++ (b) for $\mathcal {P}_{\mathcal {E}}=0.60$ and $\epsilon _{\mathcal {P}_{\mathcal {E}}}=0.01$, a problem specification on which SParSE++ achieved the highest computational savings, with gain_*i*_=48.5*%* and gain_*t*_=46.6*%*. For the 30 initial reaction rates, SParSE computed a total of 166 estimates (332 iterations), whereas SParSE++ computed only 122 estimates (171 iterations). We can see that the estimate density in Fig. 1 a is higher than (b) near the solution surface (thin area between two green dashed lines), indicating the improved efficiency of SParSE++. The difference of 161 iterations is equivalent to a savings of over 8×10^6^ simulated trajectories.

**Table 1:** Results of SParSE and SParSE++ applied to the birth-death process

$\mathcal {P}_{\mathcal {E}}$	$\epsilon _{\mathcal {P}_{\mathcal {E}}}$	Tot. Iter.	Tot. Iter.	Time (s)	Time (s)	No. Gain	No. Loss
		SParSE	SParse++	SParSE	SParSE++		
0.40	0.01	293	175	1139.0	684.9	119	1
	0.05	189	139	685.1	517.4	53	3
	0.10	139	121	476.2	457.6	21	3
0.60	0.01	332	171	1128.7	602.2	161	0
	0.05	175	141	539.5	500.1	38	4
	0.10	129	120	378.3	421.6	9	0
0.80	0.01	242	186	728.8	629.8	60	4
	0.05	139	137	449.9	480.8	3	1
	0.10	110	110	342.2	382.7	2	2
0.010	0.001	279	244	3943.5	3656.6	44	9

The first column denotes the target probability, the second column absolute error tolerance, the third column the total number of SParSE samples computed for the 30 initial parameter sets, the fourth column the total number of SParSE++ samples computed for the 30 initial parameter sets, the fifth the total number of iterations gained by running SParSE++ compared to SParSE, and the sixth the total number of iterations lost by running SParSE++ compared to SParSE. *N*_*S*_=5×10^4^ for all configurations except $\mathcal {P}_{\mathcal {E}} = 0.01$, where *N*_*S*_=2×10^5^

**Fig. 2:**
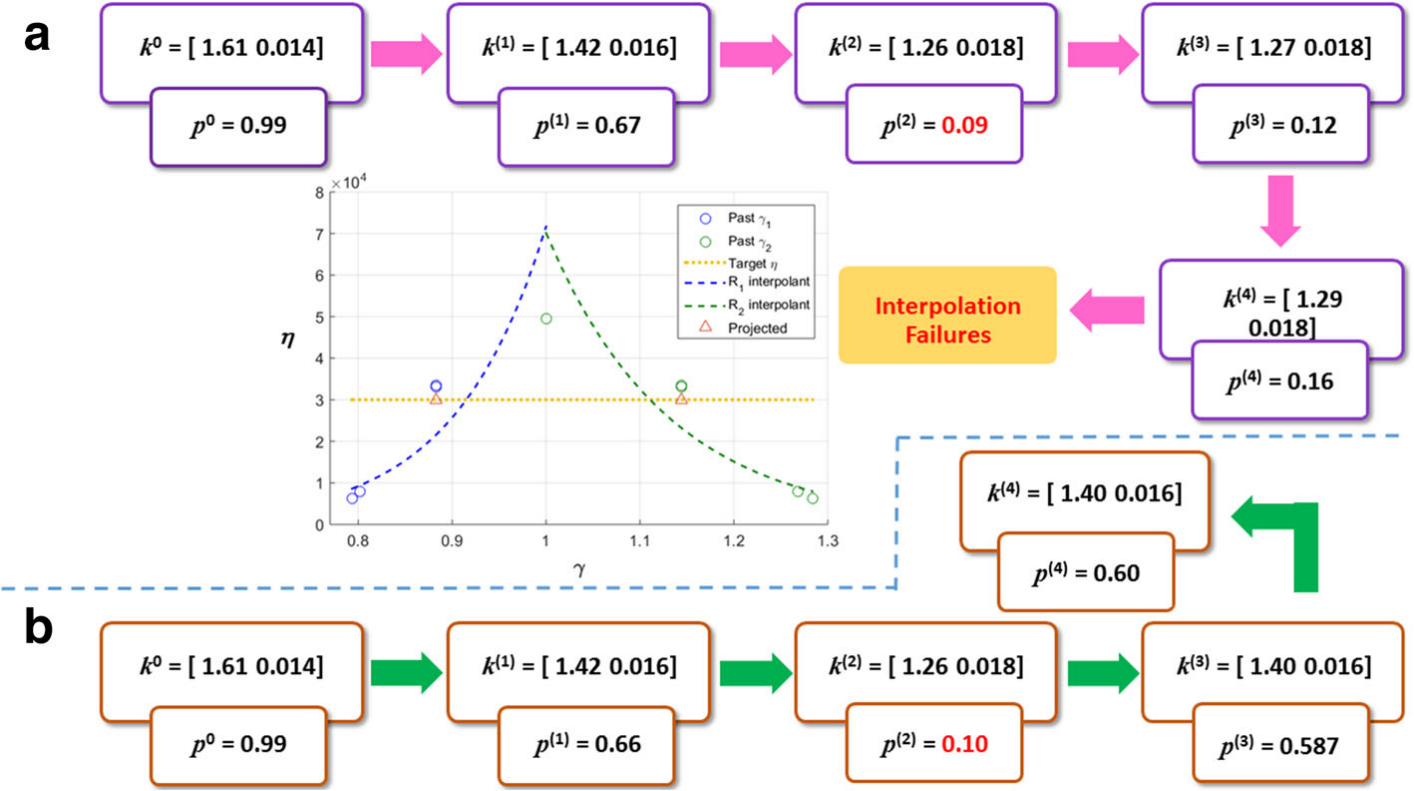
Flow chart of SParSE simulation on the birth-death process with $\mathcal {P}_{\mathcal {E}} = 0.60, \ \epsilon _{\mathcal {P}_{\mathcal {E}}} = 0.01$, and *k* ^0^= [ 1.6058 0.01401] for SParSE (**a**) and SParSE++ (**b**)

Roh and Eckhoff [16] reports that two of the thirty SParSE samples, $\boldsymbol {k^{0}_{3}} = [1.606 \; 0.0140]$ and $\boldsymbol {k}^{0}_{27} = [1.684 \; 0.0148]$ (subscript representing the index of initial reaction rates), failed to converge in the interpolation stage for $\mathcal {P}_{\mathcal {E}} = 0.60$ and $\epsilon _{\mathcal {P}_{\mathcal {E}}} = 0.01$. The reason for the failure in both cases is due to the poor agreement between past parameter estimates and their corresponding exponential interpolants. Specifically, the parameters computed by the inverse biasing method over-perturbed the system and yielded an estimate far from $\mathcal {P}_{\mathcal {E}}$. Figure 2 a summarizes the progression of SParSE with $\boldsymbol {k^{0}_{3}}$. Qualitatively, the behavior with $\boldsymbol {k}^{0}_{27}$ is similar and is thus omitted from illustration. Each over-lapping rectangular pair lists the reaction rates at stage *l* (noted as superscript) and its corresponding estimate $\hat {p}^{(l)}$. The large amount of change in $\hat {p}^{(2)}$ (highlighted in red in the Figure) from $\hat {p}^{(1)}$ despite the absolute magnitude of change in ***k*** ^(2)^ being similar to the iteration prior indicates that the probability shifts rapidly around ***k*** ^(2)^. Because $\boldsymbol {\hat {p}}^{(2-4)}$ are far from $\mathcal {P}_{\mathcal {E}}$ and ***γ*** ^(2−4)^ are obtained by the inverse biasing method, the resulting interpolant is poor and SParSE is unable to find ***k*** ^∗^. This problem is resolved in SParSE++ by applying weighted average leaping prior to entering the interpolation stage. Instead of continuing with the remaining biasing parameter candidates as in SParSE, i.e., computing $\boldsymbol {\hat {p}}^{(3-4)}$, SParSE++ stops the multi-level CE method, as both under- and over-perturbing estimates are obtained. Using the biasing parameters computed in Algorithm 2, SParSE++ places the third estimate $\hat {p}^{(3)}$ at 0.587, near $\mathcal {P}_{\mathcal {E}} = 0.60$ (Fig. 2 b). When the SParSE++ interpolation stage begins, the interpolant without the most under-perturbing estimate is chosen, as its *R* ^2^ score is highest. The removal of this outlier significantly improves the interpolant quality, and SParSE++ reaches the solution hypersurface in the first interpolation stage (i.e., ***k*** ^∗^←***k*** ^(4)^).

### Reversible isomerization model

Our second example is a reversible isomerization model, which is defined as follows:

$$\begin{array}{*{20}l} A & \stackrel{k_{1}}{\rightarrow} B,& 0.1 \leq & k_{1} \leq 0.3 \\ B & \stackrel{k_{2}}{\rightarrow} A, & 0.3 \leq & k_{2} \leq 1.0,\\ \end{array} $$

with **x**_0_=[100 0], i.e., all molecules are initially in the *A* form. The target event $\mathcal {E}$ is set to the population of isomer *B* reaching 30 before *t*_*f*_=10.

**Fig. 3:**
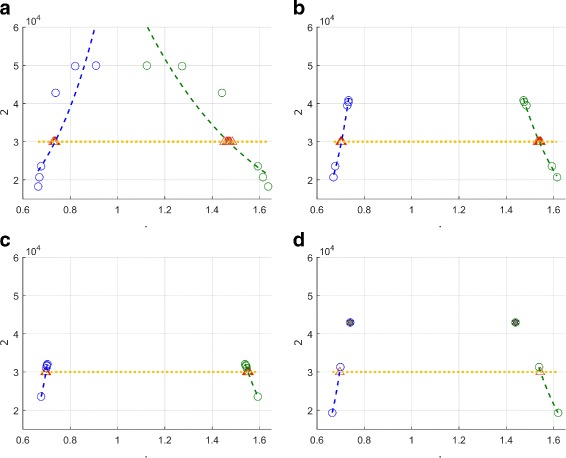
Interpolation comparison between SParSE and SParSE++ for the reversible isomerization model with $\mathcal {P}_{\mathcal {E}} = 0.60, \ \epsilon _{\mathcal {P}_{\mathcal {E}}} = 0.01$, and ***k*** ^0^=[0.2559 0.3858]. Sub-figures **a**-**c** display three successive interpolation attempts made by SParSE, where the solution hypersurface is reached by the ninth candidate biasing parameter set. Sub-figure **d** represents interpolation by SParSE++, whose first output successfully finds *k* ^∗^. *Blue* and *green circles* denote counters ($\hat {p} \cdot N_{S}$) from past biasing parameters for *R*_1_ and *R*_2_, respectively. *Blue* and *green* dashed lines represent interpolants corresponding to past counters. *Yellow horizontal line* is the target counter ($\mathcal {P}_{\mathcal {E}} \cdot N_{S}$). *Red triangles* represent counters corresponding to candidate biasing parameters

Results from 10 test cases are given in Table 2. Although SParSE achieved 100% convergence for the first nine cases, one of the 30 reaction rates for $\mathcal {P}_{\mathcal {E}}=0.95$ failed to converge [16]. In contrast, SParSE++ attained perfect convergence for all 10 test cases, required many fewer iterations than SParSE on average, and achieved up to 34.7% in gain_*i*_ ($\mathcal {P}_{\mathcal {E}}= 0.60, \epsilon _{\mathcal {P}_{\mathcal {E}}} = 0.01$) and 59.5% in gain_*t*_ ($\mathcal {P}_{\mathcal {E}}= 0.40, \epsilon _{\mathcal {P}_{\mathcal {E}}} = 0.01$). The largest gain_*i*_ achieved for a single set of initial reaction rates is 9 iterations; two reaction rates ($\boldsymbol {k^{0}_{5}}= [0.2494 \; 0.6709]$ and $\boldsymbol {k}^{0}_{26} = [0.2559 \; 0.3858]$) accomplished this for $\mathcal {P}_{\mathcal {E}} = 0.60$ and $\epsilon _{\mathcal {P}_{\mathcal {E}}} = 0.01$. For each of these two sets, SParSE required 9 iterations of interpolation before reaching the solution hypersurface. The reason SParSE employed such a high number of interpolations is the same reason the two reaction rates from the birth-death process failed to converge: past estimates from the inverse biasing method did not form an exponential trend. Running Algorithm 2 in SParSE++ eliminated this problem and required one and zero iterations of interpolation for $\boldsymbol {k}^{0}_{26}$ and $\boldsymbol {k^{0}_{5}}$, respectively. Figure 3 compares the states at which interpolation is initialized for the two methods with $\boldsymbol {k}^{0}_{26}$. Sub-figures (a)–(c) illustrate three successive interpolants computed by SParSE, while sub-figure (d) illustrates the behavior of SParSE++. When exponential interpolation is initiated for the first time (Fig. 3 a), we see that past estimates do not form a smooth trend that can be well-characterized by a single exponential function. After exhausting all candidate biasing parameters from the first interpolant, another interpolation is initiated (Fig. 3 b), this time exhibiting a much smoother trend and narrower range of estimates (i.e., ***η*** values are much closer to $\eta ^{*} = \mathcal {P}_{\mathcal {E}} \cdot N_{S}$). However, the candidate biasing parameters from the second interpolation stage still over-perturbed the system more than the allowed error tolerance $\epsilon _{\mathcal {P}_{\mathcal {E}}} = 0.01$, and a third interpolation stage was required. The first candidate from the third interpolant satisfied Eq. (2), and SParSE found ***k*** ^∗^ after computing a total of 9 estimates from interpolation. In contrast, SParSE++ converged to the solution hypersurface with the first candidate biasing parameters from interpolation (Fig. 3 d). Although the range of ***η*** in (d) is similar to that of SParSE in (b) when interpolation is initiated, the range used to compute the final interpolant is similar to that of SParSE in (c). This is because the interpolant yielding the highest *R* ^2^ score was obtained by removing the most over-perturbing parameters. Furthermore, this removal was possible because the estimate computed with biasing parameters from weighted average leaping is very close to, but slightly above, the target probability.

**Table 2:** Results of SParSE and SParSE++ applied to the reversible isomerization model

$\mathcal {P}_{\mathcal {E}}$	$\epsilon _{\mathcal {P}_{\mathcal {E}}}$	Tot. Iter.	Tot. Iter.	Time (s)	Time (s)	No. Gain	No. Loss
		SParSE	SParse++	SParSE	SParSE++		
0.40	0.01	299	208	3133.8	1269.3	93	2
	0.05	210	172	1758.9	1144.3	40	2
	0.10	175	164	1549.3	1042.6	15	4
0.60	0.01	349	228	2634.2	1266.6	125	4
	0.05	233	187	1762.5	1055.7	46	0
	0.10	188	175	1510.8	941.4	17	4
0.80	0.01	319	247	2199.7	1384.6	74	2
	0.05	227	206	1415.7	1054.3	24	3
	0.10	190	188	1330.9	979.2	7	5
0.95	0.005	316	306	3539.8	2678.5	25	15

Column identities match those of Table 1. *N*_*S*_=5×10^4^ for all configurations except $\mathcal {P}_{\mathcal {E}} = 0.95$, where *N*_*S*_=10^5^

### SIRS disease transmission system

Our third example is a Susceptible-Infectious-Recovered-Susceptible (SIRS) disease transmission system, which consists of the following three reactions:

$$\begin{array}{*{20}l} S + I & \stackrel{\beta}{\rightarrow} 2I,& 0.005 \leq & \beta \leq 0.150 \\ I & \stackrel{\gamma}{\rightarrow} R, & 0.50 \leq & \gamma \leq 4.0\\ R & \stackrel{\omega}{\rightarrow} S, & 0.10 \leq & \omega \leq 3.0,\\ \end{array} $$

with **x**_0_=[100 1 0], where **x**=[*S* *I* *R*]. This model describes an epidemiological compartment where members of *S* become infected by members of *I*, who recover from the infection at rate *γ* and transition to *R*. Once recovered, members of *R* lose immunity at rate *ω*, and this transition from recovered to susceptible replenishes the population of *S*. The target event for this system is set to the population of *I* reaching 50 before *t*_*f*_=30. Unlike the two previous examples, this model contains a non-linear reaction *R*_2_, and there is no closed-form solution for computing ***k*** ^∗^. Therefore we use the same numerical solution obtained using the SSA in [16] to evaluate accuracy of SParSE and SParSE++ estimates.

**Fig. 4:**
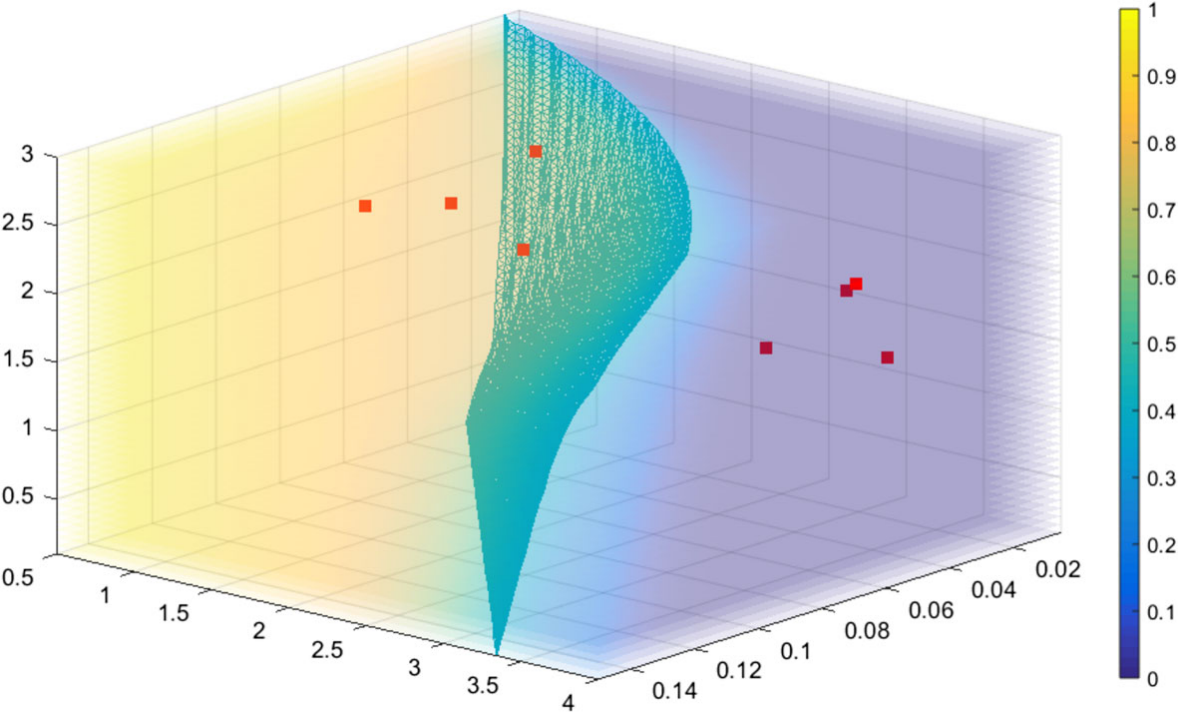
Visualization of the target event probability for SIRS model. Solution hypersurface is represented by cyan mesh grid. Eight sets of initial reaction rates that correspond to the slowest convergence in SParSE are represented by *red squares*

Table 3 summarizes the results of nine standard test cases. The biggest gain_*i*_ of 38.7*%* originates from $\mathcal {P}_{\mathcal {E}}=0.40$ and $\epsilon _{\mathcal {P}_{\mathcal {E}}} = 0.01$, where SParSE++ utilized 206 fewer iterations (equivalent to savings of 1.03×10^7^ trajectories). This is the largest gain among all examples in terms of the number of iterations saved. The substantial gain stems from a combination of the new features offered in SParSE++. To illustrate this point, we pick initial reaction rates that required more than 20 SParSE iterations to reach ***k*** ^∗^ and evaluate the corresponding performance of SParSE++ (Table 4). For each initial set of reaction rates (***k*** ^0^), SParSE iterations are divided into computation of intermediate events (IE), biasing parameters for IE (***γ***), inverse biasing from over-perturbation (OP), and interpolation (Interp). The final column (Tot) contains the sums of iterations for the given reaction rates ***k*** ^0^. For SParSE++ results, we also add the number of iterations due to leaping (Leap). Figure 4 displays the eight reaction rates in a probability plot with the solution hypersurface (cyan mesh).

**Table 3:** Results of SParSE and SParSE++ applied to SIRS disease dynamics

$\mathcal {P}_{\mathcal {E}}$	$\epsilon _{\mathcal {P}_{\mathcal {E}}}$	Tot. Iter.	Tot. Iter.	Time (s)	Time (s)	No. Gain	No. Loss
		SParSE	SParse++	SParSE	SParSE++		
0.40	0.01	532	326	4252.9	3687.7	208	2
	0.05	424	294	3602.8	3310.3	136	6
	0.10	386	247	3247.4	2863.8	141	2
0.60	0.01	328	212	826.6	589.8	117	1
	0.05	201	180	518.3	512.3	26	5
	0.10	178	156	477.2	469.0	22	0
0.80	0.01	309	306	608.9	598.1	16	13
	0.05	231	221	490.9	510.9	10	0
	0.10	183	180	399.2	436.5	3	0

Column identities match those of Table 1. *N*_*S*_=5×10^4^ for all configurations

**Table 4:** Detailed results of SParSE and SParSE++ applied to SIRS disease dynamics on eight initial reaction rates (column 1) that exhibited slowest SParSE convergence

		SParSE					SParSE++						
***k*** ^0^	$\hat {p}^{0}$	IE	*γ*	OP	Interp	Tot	IE	*γ*	OP	Leap	Interp	Tot	Gain
[0.079 1.59 2.42]	0.80	20	17	17	1	55	4	3	3	3	3	16	39
[0.081 1.07 2.28]	0.87	18	16	16	0	50	4	3	3	5	0	15	35
[0.067 3.96 2.37]	0.00	17	6	0	5	38	12	11	0	2	0	25	13
[0.064 3.31 1.71]	0.00	13	12	0	5	30	10	9	0	3	0	22	8
[0.035 1.22 2.38]	0.64	11	8	8	2	29	5	4	4	2	0	15	14
[0.040 3.59 1.52]	0.00	13	12	0	3	28	11	10	0	1	0	22	6
[0.080 2.08 2.22]	0.74	11	8	8	1	28	3	2	2	1	0	8	20
[0.031 3.16 1.84]	0.00	11	10	0	6	27	9	8	0	1	0	18	9

Initial probability estimate, number of intermediate event (IE) computations, number of biasing parameter computations, number of over-perturbation (OP) stages, and number of interpolation stages for SParSE simulations are given in columns 2-6, respectively. Column 7 contains the total number of SParSE iterations (row sum) for the initial rate in column 1. SParSE++ statistics are listed in columns 8 to 13, including the number of times the leaping method was used in column 11. The number of iterations saved by employing SParSE++ over SParSE is given in column 14 (difference between columns 7 and 13)

From Table 4 and Fig. 4, we see that some initial reaction rates are not far from $\mathcal {P}_{\mathcal {E}} = 0.40$ in absolute distance (e.g., $\hat {p}^{0} = 0.64$ and $\hat {p}^{0}=0.74$). This illustrates that the initial distance from $\mathcal {P}_{\mathcal {E}}$ cannot be reliably used to predict the speed of SParSE convergence to the solution hypersurface; if the initial estimate lies in a low-variance parameter region, the multi-level CE method may take many iterations to reach ***k*** ^∗^. We also note that the occurrence of over-perturbation alone is not highly correlated with the speed of convergence in SParSE. Three of the eight sets did not show any over-perturbation yet converged slowly to the solution hypersurface. The same holds for the number of interpolations; half of the eight reaction rates employed either 0 to 1 interpolation iterations to find ***k*** ^∗^ (Table 4). These varying behaviors demonstrate that no single modification to the SParSE algorithm would have significantly accelerated the convergence rates for all eight worst-case examples; rather, a collection of enhancements like those implemented in SParSE++ is required.

Table 4 shows that SParSE++ performed significantly better than SParSE for all eight of the initial reaction rates (Gain). For each simulation, SParSE++ saved 6 to 35 iterations, with an average savings of 18 iterations. We see that at least one of the leaping methods was employed in all eight SParSE++ simulations; Table 5 further characterizes those events. For each set of initial reaction rates, we list the number of leaping events employed by CE leaping (CE), weighted average leaping prior to interpolation (WA_Interp_), weighted average leaping in a low-signal region (WA_LowSig_), and bisection (Bisect). The final two columns sum the total numbers of leaping events (Tot) and iterations saved using SParSE++ over SParSE (Gain). Runs for all eight sets of reaction rates employed at least one round of CE-leaping, with the four slowest-converging runs employing two rounds. This illustrates the importance of CE leaping to convergence rate acceleration. Only one run utilized low-signal weighted average leaping (***k*** ^0^=[0.081 1.07 2.28]), and none utilized bisection. This behavior is expected, as these two methods are designed to handle uncommon yet challenging scenarios of rapidly changing low-signal parametric regions. Lastly, we note that only SParSE++ run on the first reaction rate set required interpolation (Table 4); for the remaining runs, applying leaping methods eliminated the need for interpolation.

**Table 5:** Detailed breakdown of SParSE++ applied to SIRS disease dynamics leaping usage on eight initial reaction rates that exhibited slowest SParSE convergence

	SParSE++ Leaping Usage					
***k*** ^0^	CE	WA_Interp_	WA_LowSig_	Bisect	Tot	Gain
[0.079 1.59 2.42]	2	1	0	0	3	39
[0.081 1.07 2.28]	2	1	2	0	5	35
[0.067 3.96 2.37]	2	0	0	0	2	13
[0.064 3.31 1.71]	2	0	0	0	2	8
[0.035 1.22 2.38]	1	1	0	0	2	14
[0.040 3.59 1.52]	1	0	0	0	1	6
[0.080 2.08 2.22]	1	0	0	0	1	20
[0.031 3.16 1.84]	1	0	0	0	1	9

Columns 2-5 list the number of times CE leaping, weighted average (WA) leaping prior interpolation, WA leaping on one-sided low signal region, and the bisection method were employed, respectively, for *k* ^0^ in column 1. The total number of leaping methods employed for each initial set of reaction rates is given in column 6. Lastly, the number of iterations saved by running SParSE++ over SParSE is listed in column 7

**Fig. 5:**
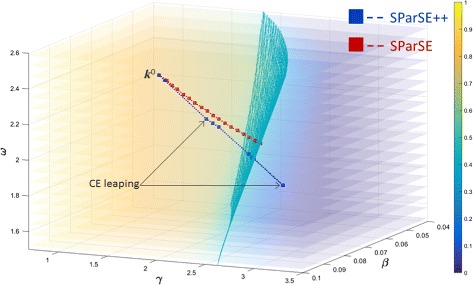
Visualization of SParSE and SParSE++ estimates converging to *k* ^∗^ for *k* ^0^=[0.079 1.59 2.42]. *Blue squares* represent SParSE++ estimates, two of which are obtained from biasing parameters computed with CE leaping. *Red squares* represent SParSE++ estimates

We note that the use of leaping often yields slightly different points in the solution hypersurface than those identified without any leaping methods. Figure 5 compares the estimate progression of SParSE and SParSE++ for ***k*** ^0^=[0.079 1.59 2.42], which corresponds to the initial reaction rates requiring the greatest number of SParSE iterations to converge (row 1 in Table 4). In this instance, SParSE ran the multi-level CE method exclusively until the very last iteration, at which point it identified $\boldsymbol {k}^{*}_{\text {SParSE}} =[\!0.065 \ 2.29 \ 2.02]$ using interpolation. In contrast, SParSE++ employed three rounds of leaping and three rounds of interpolation in addition to the multi-level CE method and obtained $\boldsymbol {k}^{*}_{\text {SParSE++}} =[0.064 \ 2.22 \ 1.92]$. We see from Fig. 5 that the first two estimates are almost identical between the two methods. This is expected, since both methods used the multi-level CE method to compute these estimates. However, SParSE++ initiates CE leaping on the third estimate upon detection of slow convergence. As a result, the third SParSE++ estimate is positioned close to but slightly below the ninth SParSE estimate. The fourth and fifth SParSE++ estimates closely parallel the 10^th^ and 11^th^ SParSE estimates, after which SParSE++ again employs CE leaping for the final estimates.

### Yeast polarization

For our final example, we modified a model of the pheromone-induced G-protein cycle in *Saccharomyces cerevisiae* given in [20] in a similar fashion as [13] so that it does not start in nor reach stochastic equilibrium within *t*_*f*_=10. Our modified system consists of seven species **x**=[*R* *L* *RL* *G* *G*_*a*_ *G*_*bg*_ *G*_*d*_] and is characterized by the following eight reactions:

$$\begin{array}{*{20}l} \emptyset &\stackrel{k_{1}}{\rightarrow} R &0.30 \leq k_{1} & \leq 0.90 \\ R &\stackrel{k_{2}}{\rightarrow} \emptyset &0.0005 \leq k_{2} & \leq 0.0015 \\ L + R &\stackrel{k_{3}}{\rightarrow} RL + L &0.0025 \leq k_{3} & \leq 0.0075 \\ RL &\stackrel{k_{4}}{\rightarrow} R &0.015 \leq k_{4} & \leq 0.045 \\ RL+G &\stackrel{k_{5}}{\rightarrow} G_{\alpha} + G_{\beta\gamma} &0.0003 \leq k_{5} & \leq 0.0030 \\ G_{\alpha} &\stackrel{k_{6}}{\rightarrow} G_{d} &0.05 \leq k_{6} & \leq 0.15 \\ G_{d}+G_{\beta\gamma} &\stackrel{k_{7}}{\rightarrow} G &0.0003 \leq k_{7} & \leq 0.0008 \\ \emptyset &\stackrel{k_{8}}{\rightarrow} RL &0.001 \leq k_{8} & \leq 0.150, \\ \end{array} $$

with **x**_0_=[70 4 0 100 0 0 0]. In this yeast polarization process, the subunit *G*_*βγ*_ is thought to play an important role of signaling for the downstream Cdc42 cycle. Here we aim to discover reaction rates that yield target event $\mathcal {E}$ of *X*(*G*_*bg*_) reaching 80 by *t*_*f*_=10 with probability $\mathcal {P}_{\mathcal {E}} = 0.60$ and error tolerance $\epsilon _{\mathcal {P}_{\mathcal {E}}} = 0.01$.

**Fig. 6:**
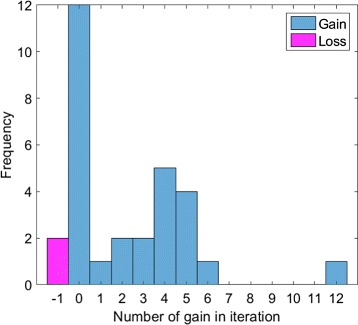
Distribution of number of iterations gained by using SParSE++ over SParSE for the yeast polarization process with $\mathcal {P}_{\mathcal {E}}=0.60$ and $\epsilon _{\mathcal {P}_{\mathcal {E}}} = 0.01$. A total of 30 data points are displayed. The *magenta bar* represents two instances of a loss of one iteration, while the remaining *blue bars* represent either no change or gains of iterations

Aggregated results from employing SParSE and SParSE++ on thirty initial reaction rates are given in Table 6. SParSE++ achieved gain_*i*_ of 19.5% and gain_*t*_ of 21.7%. We note that SParSE++ either outperformed or performed equally well as SParSE for all sets of initial reaction rates except for two, where it had a loss of only one iteration. Distribution of gain and loss in the number of total iterations per initial reaction rates is shown in Fig. 6. We see from this figure that twelve of thirty sets (40%) performed equally well with SParSE++ as with SParSE. Upon further inspection, we discovered that these twelve sets required the least number of interpolations (either 0 or 1) using SParSE. All other sets required a nonzero number of interpolations (1 to 5). For 28 out of 30 initial reaction rates, SParSE++ did not require any interpolation; weighted average leaping prior to interpolation carried the system to the solution hypersurface within the error tolerance of 0.01. We also note that SParSE++ required at most two leapings for any given set of initial reaction rates, where the majority of leaping was initiated prior to interpolation rather than from slow convergence. For systems that suffer from low stochasticity and thus slow convergence to the target event, we expect higher gains in efficiency from employing SParSE++. A list of all 30 initial reaction rates and the number of iterations required for each set by both SParSE and SParSE++ are given in Additional file 1: Appendix Section C.

**Table 6:** Results of SParSE and SParSE++ applied to the yeast polarization system

$\mathcal {P}_{\mathcal {E}}$	$\epsilon _{\mathcal {P}_{\mathcal {E}}}$	Tot. Iter.	Tot. Iter.	Time (s)	Time (s)	No. Gain	No. Loss
		SParSE	SParse++	SParSE	SParSE++		
0.60	0.01	343	275	13219.6	10350.1	69	1

Column identities match those of Table 1. *N*_*S*_=5×10^4^

In order to identify possible linear relationships among different reaction rate parameters that correspond to the solution hypersurface, we computed correlation coefficients between each pair of reactions for all ***k*** ^∗^ values from SParSE++ simulations (30 data points). We observed two correlations which were greater than 0.70 in magnitude: between reactions R1-R8 and R3-R5. Table 7 displays details from running the correlation analysis. Both identified pairs are involved in controlling the population of species *RL*. Since the ligand (*L*) population is constant in our model, the population of *RL* plays a crucial role in production of *G*_*βγ*_. The presence of too many molecules of *RL* would result in over-perturbation, while too few would result in under-perturbation with respect to the target probability ($\mathcal {P}_{\mathcal {E}} = 0.60$). The negative correlation coefficient value for R3-R5 (*r*=−0.76) implies that when *k*_3_ is set to a large value to produce many *RL* molecules, SParSE++ reduces the value of *k*_5_ to compensate for the increase in population, and vice versa. For R1-R8 (*r*=0.71), when *k*_1_ is high and many molecules of species *R* are produced, these molecules interact with *L* to produce *RL*. The resulting over-population is controlled by simultaneously increasing the degradation rate of species *RL* (*k*_8_). Reactions R1, R2, R3, R4, and R8 all directly participate in controlling the populations of the *R* and *RL* species. Running the correlation analysis identified two key reaction pairs that SParSE++ jointly perturbed to confer the target event of *G*_*βγ*_ reaching a population of 80 by *t*_*f*_=10. Such insights into the yeast polarization system may be useful for guiding future experiments in a laboratory setting.

**Table 7:** Results from applying correlation analysis on SParSE++ output of the yeast polarization model

R3-R5			
r	Lower Bound	Upper Bound	*p*-value
-0.76	-0.88	-0.55	1.0e-6
R1-R8			
r	Lower Bound	Upper Bound	*p*-value
0.71	0.48	0.85	9.4e-6

The first column denotes the correlation coefficient, the second column a lower bound from a 95% confidence interval, the third column an upper bound, and the last column the corresponding *p*-value

## Conclusions

We have developed SParSE++, a substantially more computationally efficient enhancement of SParSE for identifying parameter configurations that confer a user-defined probabilistic event. SParSE++ features novel parameter leaping methods for accelerating convergence as well as a more principled interpolation approach. Each class of leaping methods in SParSE++ has a set of prerequisite conditions. When these conditions are met, the algorithm “leaps" through parameter space, resulting in a marked reduction of the number of iterations required for convergence. This cross-entropy leaping approach, based on exponential extrapolation, permits the algorithm to converge much more rapidly for low stochasticity problems than the traditional multi-level CE method employed by SParSE. In addition, by computing a weighted average of previous estimates, SParSE++ improves the accuracy of interpolation. We note that all the merits of SParSE—high parallelizability, robustness of $\mathcal {P}_{\mathcal {E}}$ values, and concurrent updates on all reaction parameters—are retained in SParSE++.

The four examples featured in this paper demonstrate that performance gains are largest for problems requiring high accuracy. In terms of total number of iterations required, SParSE++ outperformed SParSE in 29 out of 30 test problems. For the birth-death process with $\mathcal {P}_{\mathcal {E}} = 0.80$ and $\epsilon _{\mathcal {P}_{\mathcal {E}}} = 0.10$, SParSE and SParSE++ performed equally well. This is not surprising, as most (29 out of 30) of the initial reaction rates did not require any interpolation in SParSE and converged rapidly to the solution hypersurface. For this problem configuration, each set of initial reaction rates required only 3.7 iterations on average to converge to the solution hypersurface. Similarly, SParSE++ outperformed SParSE on 25 out of 30 test problems when comparing the total runtime. For the remaining five problems, the differences in runtime are negligible (less than a minute). We also note that three sets of reaction rates that failed to converge using SParSE (two from the birth-death process and one from the reversible isomerization model) successfully reached the solution hypersurface with SParSE++.

As computational researchers continue to model events of interest in realistic biochemical systems, the need for efficient methods to identify compatible reaction rate parameters will grow. We expect that the algorithmic advancements provided by SParSE++ will fulfill this need and enable characterization of increasingly more computationally intensive biochemical events in the future.

## Additional file

Additional file 1 Appendix. In this file, we present a list of variables and definitions used in the manuscript (Section A). Detailed pseudocode for the SParSE++ driver, multi-level CE method, and inverse biasing method are given in Section B. Section C contains two tables regarding the yeast polarization process. The first table (Table 4) lists thirty randomly generated initial reaction rates that were used to run the SParSE and SParSE++ algorithms. The second table (Table 5) contains an algorithmic breakdown for each of the initial reaction rates. (PDF 234 kb)

## Abbreviations

CE: cross entropy
OP: over-perturbation
SParSE: stochastic parameter search for events
SSA: stochastic simulation algorithm
WA: weighted average

## References

[1] Komarov I, D’Souza RM (2012). Accelerating the gillespie exact stochastic simulation algorithm using hybrid parallel execution on graphics processing units. PLoS ONE.

[2] Li H, Petzold LR (2010). Efficient parallelization of the stochastic simulation algorithm for chemically reacting systems on the graphics processing unit. Int J High Perform Comput Appl.

[3] Klingbeil G, Erban R, Giles M, Maini PK (2012). Fat versus thin threading approach on gpus: Application to stochastic simulation of chemical reactions. Parallel Distributed Syst IEEE Trans.

[4] Dematté L, Prandi D (2010). Gpu computing for systems biology. Brief Bioinform.

[5] Aldinucci M, Torquati M, Spampinato C, Drocco M, Misale C, Calcagno C, Coppo M (2013). Parallel stochastic systems biology in the cloud. Brief Bioinform.

[6] Karr JR, Sanghvi JC, Macklin DN, Gutschow MV, Jacobs JM, Bolival B. Jr, Assad-Garcia N, Glass JI, Covert MW (2012). A whole-cell computational model predicts phenotype from genotype. Cell.

[7] Donovan RM, Sedgewick AJ, Faeder JR, Zuckerman DM (2013). Efficient stochastic simulation of chemical kinetics networks using a weighted ensemble of trajectories. J Chem Phys.

[8] Zwier MC, Adelman JL, Kaus JW, Pratt AJ, Wong KF, Rego NB, Suárez E, Lettieri S, Wang DW, Grabe M, Zuckerman DM, Chong LT (2015). Westpa: An interoperable, highly scalable software package for weighted ensemble simulation and analysis. J Chem Theory Comput.

[9] Reinker S, Altman R, Timmer J (2006). Parameter estimation in stochastic biochemical reactions. Syst Biol.

[10] Boys RJ, Wilkinson DJ, Kirkwood TBL (2008). Bayesian inference for a discretely observed stochastic kinetic model. Stat Comput.

[11] Toni T, Welch D, Strelkowa N, Ipsen A, Stumpf MPH (2009). Approximate bayesian computation scheme for parameter inference and model selection in dynamical systems. J R Soc Interface.

[12] Wang Y, Christley S, Mjolsness E, Xie X (2010). Parameter inference for discretely observed stochastic kinetic models using stochastic gradient descent. BMC Syst Biol.

[13] Daigle Jr. BJ, Roh MK, Petzold LR, Niemi J (2012). Accelerated maximum likelihood parameter estimation for stochastic biochemical systems. BMC Bioinformatics.

[14] Lillacci G, Khammash M. The signal within the noise: Efficient inference of stochastic gene regulation models using fluorescence histograms and stochastic simulations. Bioinformatics. 2013. doi:10.1093/bioinformatics/btt380. 10.1093/bioinformatics/btt380 23821649

[15] Liao S, Vejchodský T, Erban R. Tensor methods for parameter estimation and bifurcation analysis of stochastic reaction networks. J R Soc Interface. 2015; 12(108). doi:10.1098/rsif.2015.0233. http://rsif.royalsocietypublishing.org/content/12/108/20150233.full.pdf. 10.1098/rsif.2015.0233 PMC4528587 26063822

[16] Roh MK, Eckhoff P (2014). Stochastic parameter search for events. BMC Syst Biol.

[17] Gerardin J, Bever CA, Hamainza B, Miller JM, Eckhoff PA, Wenger EA (2016). Optimal population-level infection detection strategies for malaria control and elimination in a spatial model of malaria transmission. PLoS Comput Biol.

[18] Klünder B, Freisinger T, Wedlich-Söldner R, Frey E (2013). Gdi-mediated cell polarization in yeast provides precise spatial and temporal control of cdc42 signaling. PLoS Comput Biol.

[19] Daigle Jr BJ, Roh MK, Gillespie DT, Petzold LR (2011). Automated estimation of rare event probabilities in biochemical systems. J Chem Phys.

[20] Drawert B, Lawson MJ, Petzold L, Khammash M (2010). The diffusive finite state projection algorithm for efficient simulation of the stochastic reaction-diffusion master equation. J Chem Phys.

[21] Gillespie DT (1977). Exact stochastic simulation of coupled chemical reactions. J Phys Chem.

[22] Heinisch O, Steel RGD, Torrie JH (1962). Principles and procedures of statistics. (With special reference to the biological sciences.) Mcgraw-hill Book Company, New York, Toronto, London 1960, 481 s., 15 abb.; 81 s 6 d. Biometrische Zeitschrift.

