# Supplementary material for: SParSE++: improved event-based stochastic parameter search
Source: BMC Syst Biol. 2016 Nov 25;10:109. doi: 10.1186/s12918-016-0367-z (PMC5123426; doi:10.1186/s12918-016-0367-z)
Supplement: Additional file 1 — Appendix. In this file, we present a list of variables and definitions used in the manuscript (Section A). Detailed pseudocode for the SParSE++ driver, multi-level CE method, and inverse biasing method are given in Section B. Section C contains two tables regarding the yeast polarization process. The first table (Table 4) lists thirty randomly generated initial reaction rates that were used to run the SParSE and SParSE++ algorithms. The second table (Table 5) contains an algorithmic breakdown for each of the initial reaction rates. (PDF 234 kb) [file 12918_2016_367_MOESM1_ESM.pdf]

Appendices

A Reference Tables

Table 1: Table of definitions

Variable	Definition
$\mathcal{E}$	target event
$\mathcal{P}_{\mathcal{E}}$	target probability for $\mathcal{E}$
$\epsilon_{\mathcal{P}_{\mathcal{E}}}$	absolute error tolerance on $\mathcal{P}_{\mathcal{E}}$
$f(\mathbf{x}(t))$	event function
$\mathbf{x}_0$	$\mathbf{x}(t=0)$, initial state at time 0
$\boldsymbol{k}^0$	initial reaction rate at time 0
t_f	final simulation time
$\mathcal{T}$	$\min\Big(t_f, \Big(\min\limits_t \lvert I_{\{f(\mathbf{x}(t\lvert\boldsymbol{k}))\cap\mathcal{E}\}}=1\Big)\Big)$
$\boldsymbol{\xi}$	intermediate events
$\delta(\boldsymbol{k})$	probability distance from the target event, $\mathcal{P}_{\mathcal{E}} - \hat{p}(\boldsymbol{k})$
$\boldsymbol{\rho}(\delta)$	fractions used to compute intermediate events Eqs. $(5, 6)$
$\eta(\boldsymbol{k})$	$\sum_{i=1}^{N_S}\left[I_{\{f(\mathbf{x}_i(t\lvert\boldsymbol{k}))\cap\mathcal{E}\}}\right]$
$\hat{p}(\boldsymbol{k})$	$\frac{1}{N_S}\ \eta(\boldsymbol{k})$, numerical estimator for $\mathcal{P}_{\mathcal{E}}$

Table 2: Simulation input parameters

Parameter	Default value	Description
N_S	5×10^4	number of simulations in computing $\hat{p}(\boldsymbol{k})$
$\mathcal{L}$	10	maximum number of multilevel CE method per $\boldsymbol{k}^{(0)}$
$\mathcal{I}$	3	maximum number of exponential interpolation per $\boldsymbol{k}^{(0)}$

Table 3: Table of definitions for variables used in **Algorithms A-E**

Variable	Definition
M	number of reactions
N	number of species
$\boldsymbol{\gamma}^{\text{hist}}$	matrix of past biasing parameter values normalized with respect to $\boldsymbol{k}^0$. $\gamma^{\text{hist}}_{(i,j)}$ denotes i^{th} past biasing parameter for R_j.
$\boldsymbol{\eta}^{\text{hist}}$	vector of past η values computed. η^{hist}_i denotes i^{th} past value for η.
$\boldsymbol{\gamma}^{\text{num}}$	matrix used to accumulate the numerator in Eq. (13) in Ref. [1] matrix of size $q \times M$, where q is the number of intermediate events $\gamma^{\text{num}}_{(i,j)}$ denotes values of the matrix at row i and column j.
$\boldsymbol{\gamma}^{\text{denom}}$	matrix used to accumulate the denominator in Eq. (13) in Ref. [1] matrix of size $q \times M$, where q is the number of intermediate events $\gamma^{\text{denom}}_{(i,j)}$ denotes values of the matrix at row i and column j.
$\bar{\boldsymbol{k}}$	matrix of candidate reaction rates from exponential interpolation computed by **Algorithm 4**, vector of size $1 \times M$ $\bar{k}_j$ denotes the candidate reaction rate for j^{th} reaction.

B SParSE++ pseudocode

Algorithm A SParSE Driver

$l \leftarrow 0$
$\phi_{\text{type}} \leftarrow 1$ if $f(\mathbf{x}_0) \leq \mathcal{E}$, -1 otherwise
$\boldsymbol{k}^{(\text{cur})} \leftarrow \boldsymbol{k}^0, \quad \boldsymbol{\gamma}^0 \leftarrow$ matrix of size $1 \times M$
$\boldsymbol{\gamma}^{\text{hist}},\ \boldsymbol{\eta}^{\text{hist}} \leftarrow \emptyset$
isOverPerturbed, doLeap, lastLeap $\leftarrow$ `false`
while $l \leq \mathcal{L}$ **do**
    for $i = 1$ to number of rows in $\boldsymbol{\gamma}^{(l)}$ **do**
        $\boldsymbol{\gamma}^{(\text{l,i})} \leftarrow i^{th}$ row of $\boldsymbol{\gamma}^{(l,\cdot)}$, $\quad \boldsymbol{k}^{(\text{int},i)} \leftarrow$ Eq. (12) in Ref. [1]
        $\boldsymbol{\xi},\ \eta\left(\boldsymbol{k}^{(\text{int},i)}\right) \leftarrow$ **Algorithm B**
        $\hat{p}\left(\boldsymbol{k}^{(\text{int},i)}\right) = \frac{1}{N_S} \cdot \eta\left(\boldsymbol{k}^{(\text{int},i)}\right), \quad \delta\left(\boldsymbol{k}^{(\text{int},i)}\right) = \mathcal{P}_{\mathcal{E}} - \hat{p}\left(\boldsymbol{k}^{(\text{int},i)}\right)$
        if $|\delta\left(\boldsymbol{k}^{(\text{int},i)}\right)| < \epsilon_{\mathcal{P}_{\mathcal{E}}}$ **then**
            $\boldsymbol{k}^* \leftarrow \boldsymbol{k}^{(\text{int},i)}$

13: go to **step** 113
14: **end if**
15: $\boldsymbol{\gamma}^{\text{hist}} | \ \gamma^{\text{hist}}_{(\text{end}+1,j)} = k_j^{(\text{int},i)}/k_j^{(0)}, \ j = \{1, \cdots, M\}$
16: $\boldsymbol{\eta}^{\text{hist}} | \ \eta^{\text{hist}}_{\text{end}+1} = \eta \left(\boldsymbol{k}^{(\text{int},i)} \right)$
17: **if** lastLeap **then**
18: go to **step** 99
19: **end if**
20: **if** doLeap == `true` **then**
21: doLeap ← `false`
22: **if** Qualifies for Last leaping prior interpolation **then**
23: $l \leftarrow l+1, \ \boldsymbol{\gamma}^{(l)} \leftarrow$ **Algorithms 2**
24: lastLeap ← `true`, go to **step** 7
25: **else if** Qualifies for leaping on low-signal region **then**
26: $l \leftarrow l+1, \ \boldsymbol{\gamma}^{(l)} \leftarrow$ **Algorithms 2**
27: doLeap ← `true`, go to **step** 7
28: **else if** Qualifies for bisection **then**
29: $l \leftarrow l+1, \ \boldsymbol{\gamma}^{(l)} \leftarrow$ **Algorithms 3**
30: doLeap ← `true`, go to **step** 7
31: **end if**
32: **else**
33: **if** Qualifies for Last leaping prior interpolation **then**
34: $l \leftarrow l+1, \ \boldsymbol{\gamma}^{(l)} \leftarrow$ **Algorithms 2**
35: lastLeap ← `true`, go to **step** 7
36: **else if** Qualifies for leaping on low-signal region **then**
37: $l \leftarrow l+1, \ \boldsymbol{\gamma}^{(l)} \leftarrow$ **Algorithms 2**
38: doLeap ← `true`, go to **step** 7
39: **else if** Qualifies for bisection **then**
40: $l \leftarrow l+1, \ \boldsymbol{\gamma}^{(l)} \leftarrow$ **Algorithms 3**
41: doLeap ← `true`, go to **step** 7
42: **else if** Qualifies for CE-leaping **then**
43: $l \leftarrow l+1, \ \boldsymbol{\gamma}^{(l)} \leftarrow$ **Algorithms 1**
44: doLeap ← `true`, go to **step** 7
45: **end if**
46: **end if**
47: **if** isOverPerturbed == `false` **then**
48: **if** $\text{sgn}(\delta) \neq \phi_{\text{type}}$ **then**
49: **if** $i ==$ number of rows in $\boldsymbol{\gamma}^{(l)}$ **then**
50: **if** $\nexists$ UP data **then**
51: isOverPerturbed ← `true`
52: **else if** both UP and OP data **then**
53: go to **step** 99
54: **end if**
55: **else**
56: **continue** with $i \leftarrow i+1$
57: **end if**
58: **end if**

59: **else**
60: **if** both UP and OP data **then**
61: go to **step** 99
62: **else**
63: $\boldsymbol{\xi} \leftarrow$ **Algorithm B** # multi-level CE method with UP
64: **end if**
65: **end if**
66: **if** isOverPerturbed == `true` **then**
67: **if** $\mathrm{sgn}(\delta) == \phi_{\text{type}}$ **then**
68: **if** $i ==$ number of rows in $\boldsymbol{\gamma}^{(l)}$ **then**
69: **if** $\nexists$ UP data **then**
70: isOverPerturbed $\leftarrow$ `false`
71: $l \leftarrow l+1,\ \boldsymbol{\gamma}^{(l)} \leftarrow$ **Algorithms 2**
72: doLeap $\leftarrow$ `true`, go to **step** 7
73: **else if** both UP and OP data **then**
74: go to **step** 99
75: **end if**
76: **else**
77: **continue** with $i \leftarrow i+1$
78: **end if**
79: **else**
80: **if** both UP and OP data **then**
81: go to **step** 99
82: **else**
83: $\boldsymbol{\xi} \leftarrow$ **Algorithm C** # multi-level CE method with OP
84: **end if**
85: **end if**
86: **end if**
87: **end for**
88: $\boldsymbol{\gamma}^{\text{num}},\ \boldsymbol{\gamma}^{\text{denom}} \leftarrow$ **Algorithm D** with $\boldsymbol{k}^{(\text{cur})}$
89: $l \leftarrow l+1$
90: **if** isOverPerturbed == `false` **then**
91: $\boldsymbol{\gamma}^{(l)}|\gamma^{(l)}_{(i,j)} = \gamma^{\text{num}}_{(i,j)}/\gamma^{\text{denom}}_{(i,j)}$
92: **else**[inverse biasing]
93: $\boldsymbol{\gamma}^{(l)}|\gamma^{(l)}_{(i,j)} = \gamma^{\text{denom}}_{(i,j)}/\gamma^{\text{num}}_{(i,j)}$
94: **end if**
95: **end while**
96: **if** $l == \mathcal{L}+1$ **then**
97: Assume failure in finding $\boldsymbol{k}^{*}$ and exit
98: **end if**
99: $i \leftarrow 1$ # interpolation stage
100: **while** $i \leq \mathcal{I}$ **do**
101: $\bar{\boldsymbol{k}} \leftarrow$ **Algorithm 4**
102: $\boldsymbol{\xi},\ \eta\left(\bar{\boldsymbol{k}}\right) \leftarrow$ **Algorithm B**
103: $\hat{p}\left(\bar{\boldsymbol{k}}\right) = \frac{1}{N_S}\cdot\eta\left(\bar{\boldsymbol{k}}\right),\quad \delta\left(\bar{\boldsymbol{k}}\right) = \mathcal{P}_{\mathcal{E}} - \hat{p}\left(\bar{\boldsymbol{k}}\right)$

104: **if** $|\delta\left(\bar{\boldsymbol{k}}\right)| < \epsilon_{\mathcal{P}_{\mathcal{E}}}$ **then**
105: $\boldsymbol{k}^* \leftarrow \bar{\boldsymbol{k}}$
106: go to **step** (113)
107: **end if**
108: $i \leftarrow i + 1$
109: **end while**
110: **if** $i == \mathcal{I} + 1$ **then**
111: Assume failure in finding $\boldsymbol{k}^*$ and exit
112: **end if**
113: **return** $\boldsymbol{k}^*$

Algorithm B Intermediate Event with Under Perturbation

1: $\mathbf{v}_{\boldsymbol{k}},\ \boldsymbol{I}_{\xi} \leftarrow \overrightarrow{\mathbf{0}}$
2: **for** $i = 1$ to N_S **do**
3: $t \leftarrow 0, \quad \mathbf{x} \leftarrow \mathbf{x}_0$
4: evaluate all $a_j(\mathbf{x}),\ j = \{1, \cdots, M\}$; calculate $a_0(\mathbf{x})$
5: $\mathbf{v}_{\boldsymbol{k}}[i] \leftarrow f\left(\mathbf{x}_0\right)$
6: **while** $t \leq t_f$ **do**
7: **if** $f(\mathbf{x}) == \mathcal{E}$ **then**
8: $\boldsymbol{I}_{\xi}[i] \leftarrow 1$
9: break out of the while loop
10: **end if**
11: generate two unit-interval uniform random numbers r_1 and r_2
12: $\tau \leftarrow {a_0}^{-1}(\mathbf{x}) \ln(1/r_1)$
13: $j' \leftarrow$ smallest integer satisfying $\sum_{j=1}^{j'} a_j(\mathbf{x}) \geq r_2 a_0(\mathbf{x})$
14: $t \leftarrow t + \tau, \quad \mathbf{x} \leftarrow \mathbf{x} + \boldsymbol{\nu}_{j'}$
15: update all $a_j(\mathbf{x}),\ j = \{1, \cdots, M\}$; recalculate $a_0(\mathbf{x})$
16: **if** $\phi_{\text{type}} == 1$ **then**
17: $\mathbf{v}_{\boldsymbol{k}}[i] \leftarrow \max\left(\mathbf{v}_{\boldsymbol{k}}[i], \mathcal{E}\right)$
18: **else**
19: $\mathbf{v}_{\boldsymbol{k}}[i] \leftarrow \min\left(\mathbf{v}_{\boldsymbol{k}}[i], \mathcal{E}\right)$
20: **end if**
21: **end while**
22: **end for**
23: $\eta\left(\boldsymbol{k}\right) \leftarrow \sum_{i=1}^{N_S} \boldsymbol{I}_{\xi}[i], \quad \boldsymbol{\rho}(\delta) \leftarrow$ Eq. (10) in Ref. [1]
24: **if** $\phi_{\text{type}} == 1$ **then**
25: Sort $\mathbf{v}_{\boldsymbol{k}}$ in descending order
26: **else**
27: Sort $\mathbf{v}_{\boldsymbol{k}}$ in ascending order
28: **end if**
29: $\boldsymbol{\xi} \leftarrow$ unique elements of $\mathbf{v}_{\boldsymbol{k}}$ at indices $\lceil \boldsymbol{\rho}(\delta) \times N \rceil$
30: **return** $\boldsymbol{\xi}$ and $\eta\left(\boldsymbol{k}\right)$

Algorithm C Intermediate Event with Over Perturbation

$\mathbf{v}_{\boldsymbol{k}} \leftarrow \overrightarrow{\mathbf{0}}$ # zero vector of size N
for $i = 1$ to N_S **do**
  $t \leftarrow 0, \quad \mathbf{x} \leftarrow \mathbf{x}_0$
  evaluate all $a_j(\mathbf{x}),\ j = \{1, \cdots, M\}$; calculate $a_0(\mathbf{x})$
  $\mathbf{v}_{\boldsymbol{k}}[i] \leftarrow f(\mathbf{x}_0)$
  while $t \leq t_f$ **do**
    generate two unit-interval uniform random numbers r_1 and r_2
    $\tau \leftarrow {a_0}^{-1}(\mathbf{x}) \ln(1/r_1)$
    $j' \leftarrow$ smallest integer satisfying $\sum_{j=1}^{j'} a_j(\mathbf{x}) \geq r_2 a_0(\mathbf{x})$
    $t \leftarrow t + \tau, \quad \mathbf{x} \leftarrow \mathbf{x} + \boldsymbol{\nu}_{j'}$
    update all $a_j(\mathbf{x}),\ j = (1, \cdots, M)$; recalculate $a_0(\mathbf{x})$
    if $\phi_{\text{type}} == 1$ **then**
      $\mathbf{v}_{\boldsymbol{k}}[i] \leftarrow \max(\mathbf{v}_{\boldsymbol{k}}[i], \mathcal{E})$
    else
      $\mathbf{v}_{\boldsymbol{k}}[i] \leftarrow \min(\mathbf{v}_{\boldsymbol{k}}[i], \mathcal{E})$
    end if
  end while
end for
$\boldsymbol{\rho}(\delta) \leftarrow$ Eq. (11) in Ref. [1]
if $\phi_{\text{type}} == 1$ **then**
  Sort $\mathbf{v}_{\boldsymbol{k}}$ in descending order
else
  Sort $\mathbf{v}_{\boldsymbol{k}}$ in ascending order
end if
$\boldsymbol{\xi} \leftarrow$ unique elements of $\mathbf{v}_{\boldsymbol{k}}$ at indices $\lceil \boldsymbol{\rho}(\delta) \times N \rceil$
return $\boldsymbol{\xi}$

Algorithm D Biasing Parameter Computation

$q \leftarrow$ length of $\boldsymbol{\xi}$
$\boldsymbol{\gamma}^{\text{num}}, \boldsymbol{\gamma}^{\text{denom}} \leftarrow 0$ matrix of size $q \times M$
for $i = 1$ to N_S **do**
    $\boldsymbol{\gamma}^{\text{numi}}, \boldsymbol{\gamma}^{\text{denomi}} \leftarrow 0$ matrix of size $q \times M$
    $\boldsymbol{n},\ \boldsymbol{\lambda} \leftarrow \overrightarrow{\mathbf{0}}$ # zero vector of size M
    $\text{ind}_{IE} \leftarrow q$
    $t \leftarrow 0, \quad \mathbf{x} \leftarrow \mathbf{x}_0$
    evaluate all $a_j(\mathbf{x}),\ j = \{1, \cdots, M\}$; calculate $a_0(\mathbf{x})$
    while $t \leq t_f$ **do**
        if $f(\mathbf{x}) == \xi^{(\text{ind}_{IE})}$ **then**
            $\gamma^{\text{numi}}_{(\text{ind}_{IE},j)} \leftarrow n_j, \quad \gamma^{\text{denomi}}_{(\text{ind}_{IE},j)} \leftarrow \lambda_j, \quad j = \{1, \cdots, M\}$
            if $\text{ind}_{IE} == 1$ **then**
                break out of **while** loop in **step** ()
            else
                $\text{ind}_{IE} = \text{ind}_{IE} - 1$
            end if
        end if
        generate two unit-interval uniform random numbers r_1 and r_2
        $\tau \leftarrow {a_0}^{-1}(\mathbf{x}) \ln(1/r_1)$
        $j' \leftarrow$ smallest integer satisfying $\sum_{j=1}^{j'} a_j(\mathbf{x}) \geq r_2 a_0(\mathbf{x})$
        $t \leftarrow t + \tau, \quad \mathbf{x} \leftarrow \mathbf{x} + \boldsymbol{\nu}_{j'}$
        update all $a_j(\mathbf{x}),\ j = (1, \cdots, M)$; recalculate $a_0(\mathbf{x})$
        $n_{j'} \leftarrow n_{j'} + 1, \quad \lambda_j \leftarrow \lambda_j + a_j(\mathbf{x}) \cdot \tau, \quad j = (1, \cdots, M)$
    end while
    $\gamma^{\text{num}}_{(i,j)} \leftarrow \gamma^{\text{num}}_{(i,j)} + \gamma^{\text{numi}}_{(i,j)}, \quad i = \{1, \cdots, q\},\ j = \{1, \cdots, M\}$
    $\gamma^{\text{denom}}_{(i,j)} \leftarrow \gamma^{\text{denom}}_{(i,j)} + \gamma^{\text{denomi}}_{(i,j)}, \quad i = \{1, \cdots, q\},\ j = \{1, \cdots, M\}$
end for
return $\boldsymbol{\gamma}^{\text{num}}, \boldsymbol{\gamma}^{\text{denom}}$

C Detailed breakdown of yeast polarization simulation results

Table 4: Randomized initial reaction rates in yeast polarization process.

$\boldsymbol{k}_0^i$	R_1	R_2	R_3	R_4	R_5	R_6	R_7	R_8
1	0.4098	0.0014	0.0073	0.0259	0.0025	0.1127	0.0008	0.0327
2	0.3924	0.0013	0.0070	0.0274	0.0016	0.1322	0.0005	0.0667
3	0.6666	0.0005	0.0073	0.0410	0.0016	0.1173	0.0006	0.0050
4	0.8889	0.0010	0.0065	0.0338	0.0019	0.1169	0.0006	0.1489
5	0.4782	0.0009	0.0039	0.0363	0.0018	0.0599	0.0004	0.1240
6	0.7753	0.0008	0.0065	0.0449	0.0016	0.0573	0.0007	0.0939
7	0.8281	0.0011	0.0060	0.0154	0.0005	0.0969	0.0003	0.0335
8	0.5927	0.0007	0.0072	0.0238	0.0006	0.1464	0.0006	0.0789
9	0.7380	0.0006	0.0067	0.0310	0.0012	0.1269	0.0008	0.0636
10	0.7985	0.0009	0.0073	0.0355	0.0023	0.0782	0.0006	0.0742
11	0.3897	0.0009	0.0030	0.0277	0.0015	0.1263	0.0007	0.0903
12	0.6867	0.0006	0.0046	0.0395	0.0014	0.1420	0.0003	0.1252
13	0.8952	0.0007	0.0025	0.0436	0.0007	0.0770	0.0006	0.0917
14	0.8144	0.0013	0.0030	0.0445	0.0029	0.1344	0.0006	0.1344
15	0.7801	0.0008	0.0046	0.0387	0.0027	0.1467	0.0008	0.0848
16	0.3531	0.0013	0.0033	0.0184	0.0017	0.1221	0.0005	0.0072
17	0.6782	0.0012	0.0035	0.0363	0.0012	0.1452	0.0005	0.1367
18	0.5282	0.0008	0.0057	0.0164	0.0027	0.1029	0.0007	0.0808
19	0.3100	0.0009	0.0061	0.0420	0.0014	0.1332	0.0003	0.0777
20	0.8252	0.0014	0.0061	0.0407	0.0017	0.1449	0.0005	0.1442
21	0.7305	0.0011	0.0051	0.0230	0.0007	0.0788	0.0004	0.0539
22	0.4745	0.0006	0.0039	0.0226	0.0024	0.1390	0.0004	0.0841
23	0.6115	0.0014	0.0052	0.0424	0.0023	0.0652	0.0005	0.0512
24	0.3870	0.0008	0.0071	0.0195	0.0006	0.1288	0.0007	0.0165
25	0.3435	0.0005	0.0057	0.0416	0.0011	0.1323	0.0004	0.0458
26	0.4072	0.0012	0.0069	0.0202	0.0004	0.0977	0.0005	0.0102
27	0.3112	0.0011	0.0053	0.0410	0.0008	0.1232	0.0003	0.0121
28	0.8563	0.0009	0.0049	0.0189	0.0026	0.0976	0.0005	0.0736
29	0.3027	0.0013	0.0027	0.0355	0.0009	0.1128	0.0007	0.0167
30	0.7023	0.0012	0.0046	0.0390	0.0003	0.0598	0.0003	0.0434

Table 5: Results of SParSE and SParSE++ applied to the yeast polarization system for 30 randomized initial reaction rates. First column denotes the index of initial reaction rates from Table 4. IE denotes the number of iterations required for Intermediate Events, γ for computation of biasing parameters, OP for Over Perturbation, Interp for Interpolation, and Leap for Leaping. The last column denotes the difference in iterations employed between SParSE and SParSE++.

$\boldsymbol{k}_0^i$	SParSE					SParSE++						Iter. Gain
i	IE	γ	OP	Interp	Tot	IE	γ	OP	Leap	Interp	Tot	
1	7	4	4	6	21	2	1	1	2	3	9	12
2	5	3	3	5	16	4	3	3	1	0	11	5
3	3	2	2	0	7	3	2	2	1	0	8	-1
4	4	2	2	0	8	3	2	2	1	0	8	0
5	4	3	0	0	7	4	3	0	0	0	7	0
6	3	2	0	1	6	3	2	0	1	0	6	0
7	7	6	0	1	14	6	5	0	2	0	13	1
8	6	5	0	5	16	6	5	0	1	0	12	4
9	2	1	0	1	4	1	0	0	0	0	1	3
10	5	2	2	1	10	3	2	2	1	0	8	2
11	6	5	0	1	12	6	5	0	1	0	12	0
12	3	2	0	1	6	3	2	0	1	0	6	0
13	8	7	0	4	19	8	7	0	1	0	16	3
14	4	3	0	1	8	4	3	0	1	0	8	0
15	1	0	0	0	1	1	0	0	0	0	1	0
16	5	4	0	5	14	5	4	0	1	0	10	4
17	5	4	0	5	14	5	4	0	0	0	9	5
18	5	2	2	4	13	3	2	2	1	0	8	5
19	3	1	1	2	7	2	1	1	1	0	5	2
20	5	2	2	5	14	3	2	2	1	0	8	6
21	7	6	0	1	14	7	6	0	1	0	14	0
22	3	2	0	1	6	3	2	0	1	0	6	0
23	3	2	0	1	6	3	2	0	1	0	6	0
24	7	6	0	4	17	6	5	0	2	0	13	4
25	4	3	0	1	8	4	3	0	1	0	8	0
26	9	8	0	5	22	8	7	0	2	0	17	5
27	5	4	0	5	14	5	4	0	1	0	10	4
28	2	1	0	1	4	2	1	0	1	0	4	0
29	8	7	0	0	15	8	7	0	1	0	16	-1
30	8	7	0	5	20	8	7	0	1	0	16	4

References

[1] Roh, M.K., Eckhoff, P.: Stochastic parameter search for events **8**(1), 126. doi:10.1186/s12918-014-0126-y. 25380984. Accessed 2015-06-17
